# Statin effect on arrhythmogenic cardiomyopathy disease progression (SEARCH): Randomized clinical study protocol

**DOI:** 10.1371/journal.pone.0332876

**Published:** 2025-09-29

**Authors:** Elena Sommariva, Melania Lippi, Flavia Bruttini, Francesco Cannata, Andrea Baggiano, Marco Schiavone, Gaia Salina, Mariano Sabatino, Giulia Vettor, Rita Sicuso, Francesca Pizzamiglio, Valentina Ribatti, Riccardo Maragna, Corrado Carbucicchio, Francesca DeLuca, Valentina Catto, Ada Iezzi, Emanuela Omodeo Salè, Yari Valeri, Alessandro Barbarossa, Martina Caiazza, Emanuele Monda, Giulia Frisso, Felice Borrelli, Alessio Gasperetti, Annalisa Turco, Leonardo De Luca, Valentina D. A. Corino, Arianna Galotta, Alice Bonomi, Giulio Pompilio, Gianluca Pontone, Manuela Muratori, Giuseppe Limongelli, Raffaella Lombardi, Michela Casella, Claudio Tondo

**Affiliations:** 1 Centro Cardiologico Monzino IRCCS, Milano, Italy; 2 Università degli Studi di Milano Bicocca, Department of Medicine and Surgery, Milano, Italy; 3 Politecnico di Milano, Department of Electronics, Information and Bioengineering, Milano, Italy; 4 IEO Istituto Europeo di Oncologia IRCCS, Milano, Italy; 5 Azienda Ospedaliera Universitaria delle Marche, Ancona, Italy; 6 Department of Clinical Specialties and Odontostomatology, Università Politecnica delle Marche, Ancona, Italy; 7 Department of Translational Medical Sciences, Università Luigi Vanvitelli, Inherited and Rare Cardiovascular Diseases, Azienda Ospedaliera dei Colli, Monaldi, Napoli, Italy; 8 Università degli Studi di Napoli Federico II, Department of Advanced Biomedical Sciences, Napoli, Italy; 9 CEINGE-Biotecnologie Avanzate Franco Salvatore, Napoli, Italy; 10 Department of Medicine, Johns Hopkins University, Baltimore, Maryland, United States of America; 11 Fondazione IRCCS Policlinico San Matteo, Pavia, Italy; 12 Department of Biomedical, Surgical and Dental Sciences, Università degli Studi di Milano, Milano, Italy; PLOS: Public Library of Science, UNITED KINGDOM OF GREAT BRITAIN AND NORTHERN IRELAND

## Abstract

Arrhythmogenic cardiomyopathy (ACM) is an inherited cardiac disorder that predisposes affected individuals, especially young patients, to malignant arrhythmias, sudden cardiac death, and heart failure. The disease is characterized by myocardial atrophy and fibro-fatty replacement, predominantly affecting the right ventricle. Current pharmacological treatments primarily aim to alleviate symptoms by addressing arrhythmias and heart failure. These approaches are often complemented by invasive interventions such as implantable cardioverter defibrillators (ICDs) and radiofrequency ablations. However, none of these strategies effectively halts disease progression, highlighting the urgent need for novel disease-modifying therapies. We recently demonstrated that elevated plasma levels of oxidized low-density lipoprotein (oxLDL) correlate with more advanced stages of ACM in patients. Moreover, treatment with atorvastatin, which reduces oxLDL levels, prevented disease manifestation in a mouse model of ACM. Based on these findings, we hypothesize that statins may attenuate disease progression in ACM patients not only through their lipid-lowering effects, but also via pleiotropic actions such as antioxidant, anti-inflammatory, and autonomic modulation. To test this hypothesis, we designed SEARCH (Statin Effect on ARrhythmogenic CardiomyopatHy), an investigator-initiated, multicenter, prospective, randomized, double-blind, placebo-controlled clinical trial, aimed at evaluating the efficacy of atorvastatin in preventing ACM progression (NCT06922994). A total of 102 patients meeting ACM diagnostic criteria will be enrolled and randomized in a 1:1 ratio to receive either atorvastatin 80 mg/die or placebo for 18 months. The primary outcome will be the change in right ventricular global longitudinal strain, a sensitive echocardiographic measure of ventricular function, from baseline to 18 months. Secondary outcomes will include changes in arrhythmic burden, electrocardiography parameters, additional structural and functional cardiac indices, and circulating biomarkers. Tertiary and exploratory outcomes include the validation of risk scores for ACM progression and the identification of variables predicting the best responders to atorvastatin. Participants will undergo a comprehensive evaluation at baseline, 9 months, and 18 months, including cardiology visits, echocardiography, electrocardiography, blood testing, ICD or loop recorder interrogation, and cardiac magnetic resonance imaging (at enrollment and at 18 months only). Additional safety assessments and telephone follow-ups will be conducted throughout the study to monitor treatment adherence and potential adverse events. The SEARCH trial is expected to generate the first clinical evidence on the efficacy of atorvastatin in slowing ACM progression, thereby addressing a major unmet therapeutic need. The findings will shape the design of future large-scale studies and may pave the way for a novel, disease-modifying treatment strategy to improve outcomes and quality of life for patients with ACM.

## Introduction

Arrhythmogenic Cardiomyopathy (ACM) is a rare genetic disorder characterized by cardiac dysfunctions and arrhythmias. Throughout the course of the pathology, cardiac tissue progressively undergoes fibro-fatty replacement, accompanied by dilation, kinetic abnormalities and contractile impairments, ultimately contributing to the occurrence of malignant arrhythmias and sudden cardiac death [1]. ACM mainly affects the right ventricle (RV), but biventricular and left-dominant forms of the disease have been increasingly recognized [2].

ACM affects ~90,000 people in Europe (prevalence of 1:5000), mainly young individuals and athletes, with a dramatic impact on health and quality of life [3]. It affects men more frequently than women, with a ratio ranging from 1.3 to 2.4 [4–6]. Clinical manifestations mainly develop between the second and the fourth decade of life [6] and up to 25% of sudden cardiac death in age < 30 years are caused by ACM [7–9].

ACM is a heterogeneous genetic disorder characterized by autosomal dominant inheritance, incomplete penetrance and variable expressivity [10]. In about the 30–50% of cases, patients bear a heterozygous pathological mutation responsible for disease onset [6,11]. The recognized mutations mainly affect desmosomal genes (e.g., *PKP2, JUP, DSC2, DSG2, DSP*), but also non-desmosomal genes are involved (e.g., *TMEM43, DES, LMNA, PLN* and *FLNC*) [12,13]. However, in around half of patients, the genetic aetiology remains unknown [10].

Since their publication in 2010, the diagnostic criteria revised by Marcus et al., which encompass structural, functional, histological, electrical and genetic alterations, have been widely adopted by clinicians [14]. More recently, as a consequence of increased evidence of left ventricular (LV) and biventricular forms, Padua criteria have been proposed as an alternative method for the diagnosis [15].

To date, no aetiologic treatment capable of effectively limiting disease progression is available to patients. Current medications mainly target symptoms, and include β-blockers, anti-arrhythmic and guideline directed medical therapy drugs for heart failure (HF). Implantable cardioverter defibrillators (ICDs), catheter ablations and heart transplantation are invasive procedures that are often required in early or advanced stages of the disease [16,17]. This scenario emphasizes the compelling clinical need for novel and readily available therapeutic strategies to hamper disease progression.

The clinical presentation is quite heterogenous among patients, with high intra- and inter-family variability [18]. Recognized risk factors worsening disease progression include genetics, with risk varying depending on the specific gene involved [19–22] or by the presence of multiple pathogenic variants [23], vigorous physical activity [24], male sex [5] and frequent acute inflammatory phases [25]. Additionally, preclinical results obtained in our laboratory demonstrated that increased levels of circulating oxidized LDL (oxLDL) are associated to more severe phenotypes, encompassing increased cardiac lipid accumulation, ventricular dysfunction and augmented arrhythmic burden in ACM patients, thus representing a potential target for pharmacological strategies [26]. The contribution of oxLDL to ACM phenotype was further confirmed by *in vitro* and *in vivo* experiments in disease-relevant models, such as human cardiac mesenchymal stromal cells obtained from ACM heart biopsies and heterozygous *Pkp2* knock-out mice. Furthermore, *in vivo* experiments demonstrated the efficacy of atorvastatin administration in preventing cardiac fat accumulation and improving cardiac morphology and function.

Atorvastatin is a 3-hydroxy-3-methylglutaryl coenzyme A reductase inhibitor, widely known for its ability to reduce plasma cholesterol levels by inhibiting endogenous cholesterol synthesis [27]. In addition to their lipid-lowering properties, statins exert a range of pleiotropic effects, including antioxidant and anti-inflammatory activity, modulation of the sympathetic nervous system, interference with DKK-1 and the WNT signaling pathway, and anti-arrhythmic effects [28–32]. The combination of these actions is expected to contribute to slow down ACM progression by acting synergistically with cholesterol-lowering mechanisms.

Based on our findings and on literature data, we therefore propose the use of atorvastatin as an aetiologic therapeutic option that may have beneficial effects in moderating disease severity in ACM patients.

## Materials and methods

### Ethics statement

The study complies with the standards for ethics of experimentation and research integrity outlined in the Declaration of Helsinki, in accordance with the Regulation (EU) no 536/2014 and all relevant local regulations, the current International Conference on Harmonization (ICH) of Technical Requirements for Registration of Pharmaceuticals for Human Use Guideline for Good Clinical Practice (GCP). SEARCH has been approved by AIFA (Provvedimento EU CT 2024-514643-28-00 ID IN 26551) and by Lombardy 2 Territorial Ethics Committee (L2-127).

### Trial design

This is an independent, multicenter, prospective, randomized, double-blind, placebo-controlled phase II clinical trial.

**Fig 1:**
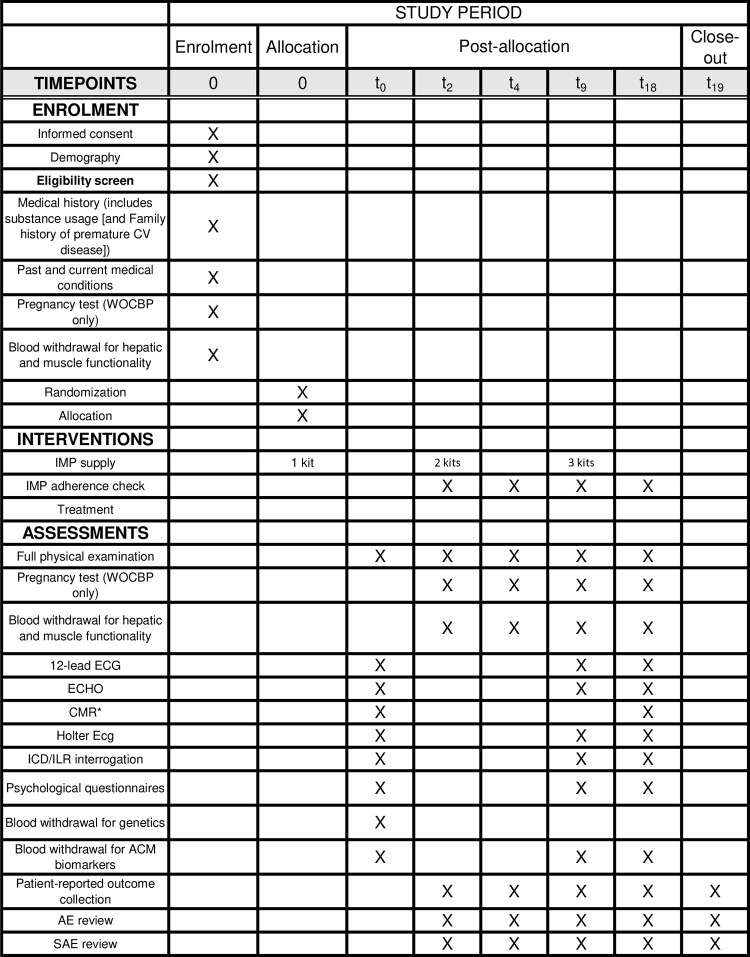
SPIRIT schedule. Resonance; CV, Cardiovascular; ECG, Electrocardiogram; ECHO, Echocardiogram; ICD, Implantable Cardioverter Device; ILR, Implantable Loop Recorder; IMP, investigation Medicinal Product; SAE, Serious adverse event; WOCBP, Women of Childbearing Potential. *If possible.

**Fig 2:**
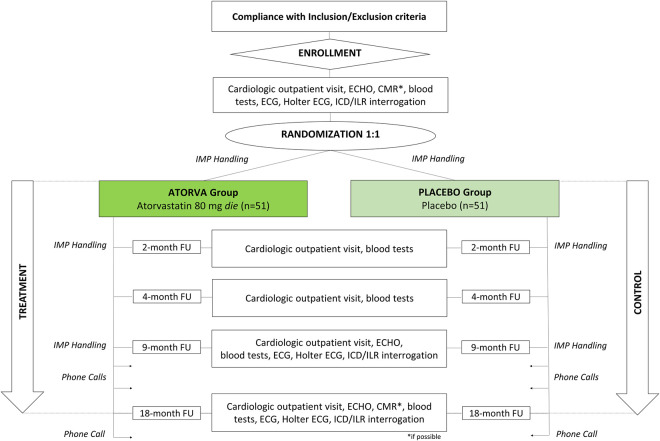
The flow chart of SEARCH trial. CMR, Cardiac Magnetic Resonance; ECG, Electrocardiogram; ECHO, Echocardiogram; FU, Follow-Up; ICD, Implantable Cardioverter Device; ILR, Implantable Loop Recorder; IMP, investigation Medicinal Product. *If possible.

The trial encompasses the following phases (Figs 1 and 2):

- Patient enrollment: patients will be enrolled after signing written informed consent and provided they meet all inclusion criteria and none of the exclusion criteria;
- Randomization and allocation: after successful screening and enrollment, participants will be randomized to receive the investigational medicinal product (IMP), and thus allocated either atorvastatin or placebo arms;
- Post Allocation period: participants will take the IMP daily for 18 months. Data collection will begin at baseline (t0 visit). During the treatment period, participants will attend in-person visits at month 2 (t2) and month 4 (t4) for safety assessments, including monitoring for any adverse events. Additional data collection and safety assessments will be performed at month 9 (t9) and month 18 (t18). Two telephone follow-up calls will be conducted at month 12 (t12) and month 15 (t15) to collect patient-reported outcomes. Unscheduled visits for safety concerns are allowed and encouraged, if necessary;
- Close-out: a final safety follow-up telephone call will be conducted approximately one month after the last dose of the IMP (t19).

### Participants

Approximately 110 participants will be screened to enroll 102 individuals who will be randomly assigned to study treatment. The goal is to obtain data from 88 evaluable participants, corresponding to an estimated total of 44 evaluable participants per treatment group.

Patients of both sexes will be enrolled, provided they meet all inclusion criteria and none of the exclusion criteria.

#### Inclusion criteria.

- At least 18 years of age at the time of signing the informed consent;
- Confirmed diagnosis of arrhythmogenic cardiomyopathy according to Marcus Task Force Criteria or Padua Criteria [2,14];
- Signed informed consent form.

#### Exclusion criteria.

- Known hypersensitivity to atorvastatin or any of its excipients;
- Moderate to severe liver disease (persistent transaminase elevation >3 × upper limit of normal);
- Muscle disease with significantly elevated creatine kinase (CK) levels (>3 × upper limit of normal);
- Left ventricular ejection fraction (EF) <35%;
- Congestive HF classified as New York Heart Association (NYHA) class III or IV;
- Known cardiomyopathy of other etiologies: ischemic, hypertrophic, idiopathic dilated, restrictive; moderate-to-severe mitral or aortic valve disease; pulmonary hypertension; congenital heart defects;
- Hypercholesterolemia requiring lipid-lowering therapy according to current guidelines [33];
- History of heart transplantation;
- Estimated life expectancy <2 years;
- Any other medical condition that, in the investigator’s opinion, places the patient at risk, renders the patient unreliable, or may interfere with study participation or completion;
- Use of potent Cytochrome P450-3A4 (CYP3A4) inhibitors, including erythromycin, clarithromycin, azole antifungals (e.g., itraconazole, posaconazole, voriconazole), protease inhibitors (e.g., ritonavir, telaprevir, boceprevir), gemfibrozil, ciclosporin, danazol;
- Use of fusidic acid (an antibacterial agent).
- Use of specific hepatitis C antivirals (e.g., telaprevir, boceprevir, glecaprevir/pibrentasvir, ledipasvir/sofosbuvir);
- Use of any lipid-lowering medications, including statins (atorvastatin, fluvastatin, lovastatin, pravastatin, rosuvastatin, simvastatin), cholesterol absorption inhibitors (ezetimibe), bile acid sequestrants (cholestyramine, colestipol), Proprotein Convertase Subtilisin/Kexin Type 9 (PCSK9) inhibitors (alirocumab, evolocumab), ATP-citrate lyase inhibitors (bempedoic acid), fibrates (gemfibrozil, fenofibrate, bezafibrate), omega-3 fatty acid ethyl esters.;
- Use of antioxidant agents primarily indicated as N-acetylcysteine.
- Participation in another clinical trial, or treatment with an investigational drug within 30 days prior to first visit (t0) or within five half-lives of the investigational drug, whichever is longer;
- Pregnant or lactating women;
- Women of childbearing potential not using adequate contraception in accordance with local regulations for clinical trial participation;
- Known alcohol or drug dependence.

#### Study sites and roles.

Recruitment and follow-up will be carried out at five Italian hospitals: Centro Cardiologico Monzino IRCCS (CCM), Milano – which is the promoter and the coordinating center -, Azienda Ospedaliero Universitaria Federico II (Università degli Studi di Napoli Federico II, Napoli), Azienda Ospedaliera Universitaria delle Marche (Università Politecnica delle Marche, Ancona), Azienda Ospedaliera dei Colli, Monaldi, Napoli, and Policlinico San Matteo IRCCS, Pavia.

CCM has designed the study and is responsible for the study coordination, data analysis, and imaging core-labs, and disease-related biomarker analyses, whereas Università degli Studi di Napoli Federico II will be responsible for conducting genetic and lipidomic analyses.

The Clinical Research Organization “Consorzio per Valutazioni Biologiche e Farmacologiche” will be responsible for monitoring and pharmacovigilance activities to confirm compliance with applicable laws, regulations, the study protocol, and standard operating procedures throughout the course of the trial.

### Intervention

The IMP consists of atorvastatin Teva Italia 80 mg film-coated tablets (an approved prescription medicine – class A – containing atorvastatin calcium salt, belonging to the therapeutic group of statin hypolipidemic agents), orally administered at a dose of 1 tablet per day, or a corresponding placebo.

Atorvastatin was purchased from commercial sources, while the placebo was manufactured by an authorized pharmaceutical company in compliance with the principles and guidelines of Good Manufacturing Practice (GMP) for medicinal products.

Packaging and labeling of both atorvastatin and placebo were conducted in accordance with Regulation (EU) No 536/2014. Each participating center was supplied with an adequate quantity of the IMP, which has been securely stored at room temperature.

Characteristics of atorvastatin and placebo are summarized in Table 1.

**Table 1:** IMP characteristics.

Treatment	Atorvastatin Teva Italia	Placebo
**Dosage formulation**	Film-coated tablets, white, shaped elliptical, and coated with smooth film. The dimensions of each tablet are approximately 18.8 mm x 10.3 mm.	Film-coated tablets, white, shaped elliptical, and coated with smooth film. The dimensions of each tablet are approximately 18.8 mm x 10.3 mm.
**Unit dose strength(s)**	80 mg of atorvastatin calcium	–
**Daily dosage level(s)**	80 mg	–
**Route of Administration**	Oral	Oral
**Dosing instructions:**	One tablet/die to take with or without food indifferently	One tablet/die to take with or without food indifferently
**Packaging and Labeling**	Study treatment will be provided in a primary packaging consisting of aluminum blisters of 10 tablets each, and a secondary packaging consisting of a box of 12 blisters. Each blister and each box will be labeled as required per country requirement. Each labeled box containing labeled blisters constitutes a KIT	Placebo will be provided in a primary packaging consisting of aluminum blisters of 10 tablets each, and a secondary packaging consisting of a box of 12 blisters. Each blister and each box will be labeled as required per country requirement. Each labeled box containing labeled blisters constitutes a KIT
**Manufacturer**	Tablets: Teva Italia S.r.l. Packaging: Euromed Pharma Services S.R.L.	Tablets: Laboratorio Farmacologico Milanese S.r.l. Packaging: Euromed Pharma Services S.R.L.

### Outcomes

#### Primary outcomes.

**Fig 3:**
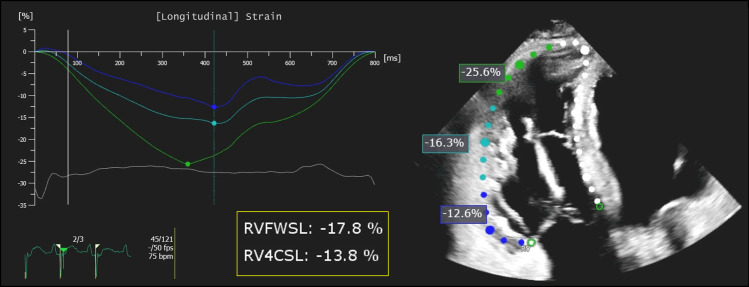
Assessment of right ventricular longitudinal strain using speckle-tracking echocardiography from an apical four-chamber view. The curves display longitudinal strain values over time for individual free-wall RV segments (basal, mid, apical). RVFWSL, right ventricular free wall strain; RV4CSL, right ventricular four-chamber longitudinal strain.

The primary objective of SEARCH trial is to assess atorvastatin efficacy in avoiding RV functional deterioration. Decay from baseline (t0) of the RV free wall longitudinal strain (RV-FWLS) will be measured by echocardiography (ECHO) at t18 and quantified in percentage (Fig 3).

#### Secondary outcomes.

Secondary objectives encompass the evaluation of atorvastatin

- efficacy to avoid electric deterioration, measured in terms of arrhythmic burden: Premature Ventricular Contraction (PVC), non-sustained and sustained ventricular arrhythmias, ventricular fibrillation, appropriate ICD shocks;
- efficacy to avoid morphological deterioration, quantified in terms of altered LV and RV dimensions, cardiac tissue composition and functions measured by both ECHO and CMR;
- efficacy in avoiding the worsening of disease biomarkers levels, including markers of cardiac damage (BNP, NTpro BNP, Troponin I, BIN1, HSP70), plasma lipids (LDL, oxLDL, lipidomics), inflammatory markers (CRP, Interleukin 6, TNF-α), fibrosis (Timp1, PIIINP, ST2, TGF-β, GAL3), oxidative stress (MDA, 4HNE), hormones (estradiol, testosterone). Biomarkers will be analyzed by the diagnostic laboratories of the participating centers using clinical-grade kits, when available in standard clinical settings. To ensure protocol standardization, biomarkers without standardized diagnostic procedures will instead be centrally analyzed at the research laboratory of Centro Cardiologico Monzino IRCCS, using available research-grade ELISA kits.
- safety, by monitoring the number and severity of adverse events and patients’ general well-being, both physical and psychological.
- effect on different ACM genetic etiologies: ACM patients without prior genetic testing will undergo whole-exome sequencing at the University of Naples Federico II. Exome capture will be performed using Agilent SureSelect Human All Exome V6, with unique barcoding for sample multiplexing. Sequencing will be conducted on an Illumina NovaSeq 6000. Variants in genes associated with ACM, as defined by the Expert Consensus Statement, will be recorded in the CRF.
- effects on specific lipid pathways: a lipidomic profile will be assessed at baseline and after treatment, including multiple lipid classes (e.g., di-/triglycerides, acylcarnitines, cholesteryl esters, ceramides). Identifying dysregulated lipids restored by treatment will help define predictive metabolic markers. Lipidomic analysis will be performed at the University of Naples Federico II using the MxP® Quant 500 XL kit (Biocrates) and mass spectrometry, enabling quantification of over 1,000 metabolites across >400 metabolic indicators and pathways.

#### Tertiary outcomes.

Tertiary objectives represent future research directions building on the prospective data collected in this trial:

- validation of risk scores for ACM progression: a retrospective study on larger ACM cohorts will be conducted, assessing the association of standard clinical parameters, symptoms, imaging data, biomarker levels, and genotypes with poor prognosis, both in terms of arrhythmias and structural degeneration, to develop a predictive model. The selected predictive variables will then be validated in the placebo arm of the SEARCH cohort at baseline (t0), by evaluating their ability to predict disease progression observed at follow-up (t18);
- prediction of differential response to atorvastatin treatment: all the above-mentioned baseline (t0) variables in the treatment arm of the SEARCH study will be analyzed in relation to atorvastatin efficacy at t18, in order to identify the best predictors of treatment response.

### Sample size

To calculate the number of participants needed for this trial, we performed a power analysis using clinical data obtained from ACM patients of CCM on which two consecutive ECHOs were performed.

This analysis estimated a sample size of 88 patients (44 per group) to observe at t18 a significant (alpha = 0.05) mean absolute difference between the two groups of 2.4 points of RV-FWLS (%), hypothesizing a standard deviation of 4.5% and with a power of 80%. The statistical test used to calculate the sample size was an independent one-sided T test. Additionally, we assumed a possible 15% drop-out rate, since adverse events and patient withdrawal need to be considered in the context of a long treatment. Considering these factors, the study will enroll 51 patients per group, for a total of 102 subjects. Assuming a screening failure of about 5%, 110 patients will be screened.

### Study stopping guidelines

The study will be terminated if a death, possibly or probably related to the IMP, occurs or if more than 15% of patients discontinue treatment due to severe adverse events (SAEs) related to the systemic use of oral IMP. A safety monitoring board is responsible for ongoing safety surveillance and can define further stopping rules or discontinuation criteria based on adverse events registered during the trial.

The study can be terminated prematurely by the promoter in case of new information leading to an unfavorable risk-benefit assessment of the IMP, or if the trial is no longer justified for clinical or ethical reasons, or if the IMP is withdrawn from the market for safety reasons.

Finally, the trial can be terminated or suspended at the request of health authorities.

### Randomization

Patients will be randomized to determine treatment allocation (atorvastatin or placebo) on top of the conventional therapy. Participants will be randomly assigned to treatment groups in a 1:1 ratio according to a randomization list. Each site will use its own independently generated randomization list. The randomization list has been generated using SAS 9.4 statistical software (version 9.4, SAS Institute, Cary, NC, USA) without stratification or blocking.

### Allocation

The randomization sequence was implemented through the assignment of centrally generated three-digit codes, securely stored at the coordinating center. The first digit of the code identifies the study site, while the second and third digits indicate the treatment allocation in a blinded format. Each consecutive eligible patient will be assigned one of these codes in a sequential manner. The allocation procedure ensures that the treatment assignment will remain concealed until the end of the study, thereby minimizing selection bias. No crossovers are permitted once treatment has been assigned.

### Implementation

The randomization sequence was generated by the coordinating center biostatisticians. Eligible participants will be enrolled by the local investigators at each recruiting center and allocated to the first available randomization code.

### Blinding

SEARCH is a double-blinded study. Until the end of the study, neither the patients nor the investigators will know which participant is receiving the drug or the placebo, except in cases of necessity. In addition, a blinded CMR and ECHO core laboratory will interpret imaging data.

To preserve blinding, a third party (Euromed Pharma Services S.r.l.) was responsible for the blinding procedures, including packaging, labeling, and distribution of the IMP to the study sites. These activities were carried out in accordance with the randomization list provided by the promoter’s statisticians, ensuring that the timing of IMP delivery to each site’s pharmacy was not influenced by the assigned treatment arm.

Atorvastatin 80 mg and placebo tablets were manufactured to be visually indistinguishable, and the labeling did not reveal the actual treatment, indicating only the randomization code.

During 18 months of treatment, investigators can break the blinding exclusively if it may compromise the patient’s health.

At the end of the 18-month treatment, an independent statistical team will analyze outcomes comparing the partially unblinded groups “A” and “B”.

In the case of quality assurance audits, reviewers will be able to access unblinded study treatment records to establish that randomization and distribution were carried out correctly.

### Statistical methods

Continuous variables will be reported as mean ± standard deviation (SD) if normally distributed, otherwise as median and interquartile range (IQR). Right-skewed variables will be log-transformed prior to analysis; if transformation is not appropriate, non-parametric methods will be used. Categorical variables will be presented as frequencies and percentages. Possible differences in baseline characteristics of ACM patients belonging to the “Atorvastatin” and “Placebo” groups will be reported in a descriptive manner. Changes in the variables of interest, measured at different timepoints, will be assessed by paired samples Student’s T test or Wilcoxon signed-rank test, and will be compared between groups by Student’s T test for independent samples or Mann Whitney’s U test, depending on data distribution. For the primary and secondary endpoints, univariate and multivariate linear regression models will be applied to estimate treatment effects, adjusting for potential confounding variables identified a priori (e.g., baseline characteristics, concomitant medications). The regression models will include group assignment as the main predictor. As proof of mechanism, Person’s or Spearman’s correlation coefficients will be calculated to explore possible relationships between endpoints and plasma oxLDL reduction. Psychological outcomes, measured via questionnaires at multiple timepoints, will be analyzed using repeated-measures ANOVA models to evaluate changes over time within and between groups. Where appropriate, paired t-tests will be used for within-subject comparisons, with correction for multiple comparisons and adjustments for confounding variables. Subgroup analyses will be conducted to explore whether treatment effects vary according to predefined factors, including concomitant medications, underlying genetic mutations, and disease stage. Adjusted analyses will involve multivariable regression models to account for potential confounding factors.

## Results

Patient enrolment started on 31 March 2025, and it is expected to finish by 31 October 2025. Participants’ information will be collected at different timepoints, accordingly to SEARCH timetable, and recorded in an electronic case report form (eCRF). Study-related paper documents will be securely archived, whereas digital files will be stored on password-secured (o access-controlled) computers to ensure utmost data security and confidentiality. The end of the trial is expected to be by half of 2027. Results will be made available through a scientific publication, once finalized.

## Discussion

This study represents one of the few prospective randomized controlled trials in ACM, and the first targeting disease aetiology. The aim of the study is to identify a pharmacological treatment effective in attenuating the progression of the disease by reducing the fatty infiltrates by a daily lipid-lowering therapy with atorvastatin 80 mg. Indeed, in ACM, fibro-fatty tissue remodelling contributes to the arrhythmic burden and it is responsible of the functional alterations that characterize disease prognosis.

The decision to propose a phase II randomized trial has been based on the knowledge of statins, which are safe commercial FDA (Food and Drug Administration)- and EMA (European Medicines Agency)-approved drugs broadly used for primary and secondary prevention of acute coronary syndrome [34–36].

In particular, atorvastatin has been chosen for its high anti-oxidant effect and for the promising results obtained in our laboratory*. In vitro* studies demonstrated the efficacy of atorvastatin in reducing levels of oxLDL and in counteracting the adipogenesis differentiation of cardiac mesenchymal stromal cells, which are crucial mechanisms driving the fatty replacement of the myocardium and the progression of ACM [26]. *In vivo* results confirmed the positive effect of atorvastatin in slowing down disease evolution, through the reduction of oxLDL levels and, consequently, fatty infiltration and RV dysfunction [26]. We expect that these lipid-lowering and anti-oxidant properties of atorvastatin will successfully ameliorate the prognosis in ACM patients.

Furthermore, we envisage that other pleiotropic effects of atorvastatin can be beneficial to ACM patients. The anti-inflammatory potential of statins has emerged from both preclinical studies [37,38] and randomized clinical trials [39,40]. The key mechanism involves the inhibition of isoprenoids formation, suppressing vascular and myocardial inflammation and redox state and improving nitric oxide availability [28]. Although it remains unclear whether it represents a primary event or a secondary response, the detrimental role of inflammation in ACM is widely recognized and its modulation through statins could contribute to reduce myocardial damage and arrhythmia in ACM patients [41]. Another documented pleiotropic effect of statins is the modulation of the sympathetic nervous activity [30,42], although the specific mechanisms are not fully known. In particular, a meta-analysis conducted by Lewandowski et al. highlighted how statins are able to reduce sympathetic central neuron system activity measured by microneurography. The lipophilia and the great ability to cross the blood-brain barrier of simvastatin and atorvastatin confer excellent potential for these statins to influence the autonomic nervous system [30]. Given the crucial impact of the adrenergic stimulation to the penetrance of the disease, the reduction of the adrenergic outflow by statin administration in ACM might represent an important contributor to risk factor reduction. In addition, a protective role of statins against the occurrence of ventricular and atrial arrhythmias have been reported [29]. The proposed mechanisms are the adjustment of the fatty acid composition of the sarcolemma, whose alteration can affect electrical conduction and excitability, and the modulation of oxidative stress, which contributes to the sarcoplasmic reticulum damage and calcium overload [29]. Thus, atorvastatin treatment might represent an opportunity to improve the patient’s overall clinical picture by concurrently exerting hypolipidemic effect, and acting on other critical aspects of ACM pathogenesis.

The safety of atorvastatin has been already established through phase I-III clinical trials [43] and an official technical datasheet is available [44]. Oral administration of atorvastatin 80 mg/die has been shown to efficiently reduce plasma levels of oxLDL in previous clinical studies [45]. The same dose was also demonstrated to be safe, with no significant differences in the incidence of adverse events compared with lower doses [46], even for treatment duration longer than 18 months [47], as we propose.

Prospective randomized pharmacological clinical trials on ACM are limited in number and mostly include arrhythmic primary outcomes, such as PVC burden, ventricular tachycardia burden, ICD intervention, and/or RV function by the evaluation of FWLS by ECHO (NCT06174220, NCT03685149, NCT03593317). Accordingly, the primary endpoint outcome we chose is RV-FWLS, while other parameters, such as arrhythmias occurrence, which is expected to decrease, as reported for other non-ischemic populations in treatment with statins [48], electrocardiographic (ECG) abnormalities, other imaging readouts, clinical signs and hematic biomarkers have been included as secondary outcomes. RV-FWLS is a clinically meaningful and measurable parameter reflecting the deformation of the RV free wall along the longitudinal axis. Compared to RV EF, it is more sensitive in detecting early systolic dysfunction and it has been shown to be associated with prognosis in various clinical conditions, including ACM [49–51]. RV-FWLS is indeed an early indicator of functional alterations, which allows the detectability of little worsening in a short time, even when EF was preserved, as reported in the literature [52], and confirmed in a CCM retrospective cohort: two ECHOs 18 months apart of 20 ACM patients were analyzed and the reduction of 2.4 points of RV-FWLS (12.8% ± 4.5) was detected (data not published), whereas differences in EF % were not significant in the same period. Furthermore, the possibility of exploiting a prognosis-associated parameter measurable by ECHO allows us to avoid the complications associated to CMR. Most ACM patients carry an ICD, which can be incompatible with CMR or generate several artifacts, making the exam difficult to be evaluated in some cases. On the other hand, results from ECHOs are often operator-dependent and the participation of different centers might highlight this limitation. Nevertheless, the involvement of the core lab should help ensure consistency in the results obtained from the five different centers and minimize any potential differences in management between hospitals. In any case, the multicenter approach should be considered advantageous for several reasons. Firstly, it offers the opportunity of including a heterogeneous population in terms of age, sex, genetic background, and environmental factors. The results will therefore be more representative of the real-world population, increasing the external validity of the study. Secondly, the involvement of five centers will facilitate the enrollment of the sample size required for adequate statistical power.

Given the rarity of ACM and the risk of patient drop-out, we defined a feasible sample size based on prior pharmacological trials in ACM (NCT03593317, NCT06174220) and a retrospective study at CCM. Several potential endpoints were excluded due to the unrealistically large sample sizes they would have required. Although 24-hour PVC count was a more achievable endpoint, we considered it suboptimal, as atorvastatin is only indirectly expected to influence arrhythmic burden, primarily through effects on RV function. Moreover, PVC count is highly variable and would demand a larger cohort. To account for the challenges of a long-term intervention, we conservatively estimated a 15% drop-out rate.

The decision to adopt an 18-month treatment period stems from the progressive yet generally slow nature of ACM in most patients. A retrospective analysis of our cohort demonstrated a measurable average progression in RV-FWLS over 18 months. Consequently, this duration represents the minimum timeframe necessary to detect a potential beneficial effect of atorvastatin treatment.

The selection of the brand (Atorvastatin TEVA) is based on its simple and smooth tablet formulation (uncoated, colourless, and without markings) making it easier to replicate as a placebo. Moreover, it is readily available and easily accessible on the Italian market.

The safety of participants is a primary concern in this study. Most exclusion criteria have been carefully selected to minimize the risk of treatment-related adverse effects by excluding patients with conditions or factors that could increase vulnerability to atorvastatin’s side effects. Throughout the trial, patients will be closely monitored approximately every three months through outpatient visits or telephone calls. These follow-ups are designed to promptly identify any AEs, serious adverse events SAEs, or suspected unexpected adverse reactions. To ensure patient safety and maintain high compliance, unscheduled visits will be arranged as needed for additional assessments or interventions. This vigilant monitoring framework aims to balance the assessment of atorvastatin’s efficacy with rigorous safety oversight.

Ultimately, this protocol can provide a framework for future research on ACM disease progression, its prediction and therapeutic strategies to mitigate it.

### Trial limitations.

This study presents limitations that should be acknowledged. First, CMR imaging may be affected by artifacts in patients with ICDs; although recent developments in artificial intelligence may help mitigate these limitations, this remains a potential source of data loss or reduced image quality.

Second, imaging and electrocardiographic data are being collected across multiple centers using different equipment and protocols (e.g., ECHO, CMR, ECG, Holter), which may introduce inter-laboratory variability. Efforts such as the involvement of a core lab are aimed at minimizing this impact.

Third, the study population is predominantly composed of male patients and athletes, and an amendment has been requested to increase the sample size, in part to address the expected imbalance across sex and phenotypes. As a result, the findings will be most representative of athletic male patients with right-dominant forms of ACM, limiting their generalizability to female patients, non-athletes. Additionally, although the primary endpoint is based on RV function, the study includes patients with left-dominant or biventricular forms, which may dilute the primary endpoint’s applicability to those phenotypes.

Finally, the emergence of updated international guidelines on the genetic classification of ACM highlights the heterogeneity of underlying genetic variants. Despite the anticipated prevalence of *PKP2* mutations in our cohort, the sample size will likely be insufficient to stratify analyses based on individual genotypes.

### Generalizability.

The multicenter design of this trial enhances its external validity by enrolling a heterogeneous population across different clinical settings, reflecting real-world variability in patient demographics, disease phenotypes, and management practices. This diversity improves the applicability of the results to routine clinical care. To further ensure the reliability of findings, the use of a placebo control is incorporated to minimize confounding factors such as patient and physician expectations, spontaneous disease fluctuations, and symptom perception. This approach not only strengthens the accuracy of efficacy assessments but also allows a more precise evaluation of atorvastatin’s side effects and its impact on patients’ well-being, which is especially relevant for the secondary endpoints.

## Other information

The trial has been registered to CTIS (ID: 26551) and to clinicaltrials.gov (NCT06922994).

The full trial protocol (S1 Appendix) and the Standard Protocol Items: Recommendations for Interventional Trials (SPIRIT) outcome checklist (S2 Appendix) can be consulted in the supplementary material.

All authors hereby declare the absence of any conflict of interests in relation to the SEARCH trial.

## Supporting information

S1 Appendix SEARCH protocol. (PDF)

S2 Appendix SPIRIT Outcome checklist [53]. (PDF)
