# Supplementary material for: Statin effect on arrhythmogenic cardiomyopathy disease progression (SEARCH): Randomized clinical study protocol
Source: PLoS One. 2025 Sep 29;20(9):e0332876. doi: 10.1371/journal.pone.0332876 (PMC12478911; doi:10.1371/journal.pone.0332876)
Supplement: S1 Appendix — (PDF) [file pone.0332876.s001.pdf]

Clinical Study Protocol
Statin Effect on ARrhythmogenic CardiomyopatHy disease progression

Protocol Code	SEARCH
Investigational Product Name/Number	Atorvastatin TEVA 80 mg
Short title	SEARCH
Sponsor Name	Centro Cardiologico Monzino, Istituto di Ricovero e Cura a Carattere Scientifico, S.p.A.
Sponsor Legal Registered Address	Via Filodrammatici 10, 20121 Milan, Italy
Sponsor Operative Address	Via Parea 4, 20138 Milan, Italy
Founds	Financed by the Italian Ministry of Health grant - PNRR-MCNT2-2023-12376978
EU CT No.	2024-514643-28-00
Version Number	3.0
Date	01.10.2024

STATEMENT OF CONFIDENTIALITY

Information in this protocol and accompanying documents contains privileged or confidential information that is the property of Centro Cardiologico Monzino, Istituto di Ricovero e Cura a Carattere Scientifico, S.p.A., Via Filodrammatici 10, 20121 Milan (CCM). It is understood that the information will not be used, divulged, or published without prior written consent of CCM, except to the extent such disclosure is required by applicable laws and regulations.

Protocol Signature Sheet and Acknowledgement

Prof. Claudio Tondo, Director of the Arrhythmology Department CCM and the Sponsor representative have discussed this protocol version and agreed that it contains all necessary details for carrying out the trial. The investigations will be performed as by the protocol except the case of medical emergency.

Problems related to this trial, should be referred to the Coordinator of the research project and holder of the PNRR-MCNT2-2023-12376978 fund, Elena Sommariva, Unit of Vascular Biology and Regenerative Medicine.

Prof. Claudio Tondo agrees to conduct and supervise the clinical study.

CCM confirms that the investigators and institutions involved in the clinical trial are to permit clinical trial-related monitoring, audits and regulatory inspections, including provision of direct access to source data and documents.

Sponsor Signature:

Signature: ______________________________ Date: 30/10/2025

(Prof. Claudio Tondo)

Investigator Protocol Approval Page

I have read this protocol and agree to conduct this trial in accordance with all stipulations of the protocol and in accordance with the Regulation (EU) no 536/2014 and all relevant local regulations, the current International Conference on Harmonization (ICH) of Technical Requirements for Registration of Pharmaceuticals for Human Use Guideline for Good Clinical Practice (GCP) and with the principles of the most recent version of the Declaration of Helsinki.

Investigator Name ____

Site Name/No ____

Address ____

____

____

Tel: ____

Investigator Signature: ____

Date ___/____/______

Study Personnel

SPONSOR
Contact Name: Centro Cardiologico Monzino, Istituto di Ricovero e Cura a Carattere Scientifico, S.p.A.
Address: via Parea 4, 20138 Milan, Italy
Tel: +390258002456
Fax: +390258002623
Mail: dirsci@cardiologicomonzino.it

COORDINATING INVESTIGATOR
Contact Name: Prof. Claudio Tondo
Position: Director of the Arrhythmology Department, Centro Cardiologico Monzino, Istituto di Ricovero e Cura a Carattere Scientifico, S.p.A.
Address: Via C. Parea 4, 20138 Milan, Italy
Tel: +39 02 5800 2275
Mail: claudio.tondo@cardiologicomonzino.it

PHARMACOVIGILANCE
Contact Name: Consorzio per Valutazioni Biologiche e Farmacologiche (CVBF)
Address: Viale Cesare Battisti 17, Pavia, Italy
Tel: +39 373 8530614
Fax: +39 080 9909321
E-mail: pharmacovigilance@cvbf.net

MONITORING
Contact Name: Consorzio per Valutazioni Biologiche e Farmacologiche (CVBF)
Address: Viale Cesare Battisti 17, Pavia, Italy
Tel: +39 0382 1475411

DATA and SAFETY MONITORING BOARD
Contact Name: Dr. Marco Scatigna, Scuola di Specialità in Farmacologia Clinica, Università degli Studi di Milano
Address: Via Santa Sofia 9,20122 Milan, Italy
Tel: +39 02 5800 2340
E-mail: marco.scatigna@unimi.it

CENTRAL LABORATORY of GENETICS and LIPIDOMICS
Contact Name: Dr. Giulia Frisso, Dipartimento di Medicina Molecolare e Biotecnologie Mediche, Università degli Studi di Napoli Federico II
Address: Via Pansini, 5, Napoli, Italy
Tel: +39 081-7462405
E-mail: giulia.frisso@unina.it

CENTRAL LABORATORY for RESEARCH BIOMARKERS
Contact Name: Dr. Elena Sommariva, Unit of Vascular Biology and regenerative therapy, CCM
Address: via Parea 4, 20138 Milan, Italy
Tel: +39 02 58002752
E-mail: elena.sommariva@cardiologicomonzino.it

IMAGING CORE LABORATORY
Contact Name: Dr. Andrea Baggiano, Unit of Peri-Operative Cardiology and Cardiovascular Imaging, CCM
Address: via Parea 4, 20138 Milan, Italy
Tel: +39 02 58002574
E-mail: andrea.baggiano@cardiologicomonzino.it

ARTIFICIAL INTELLIGENCE ANALISIS LAB
Contact Name: Prof. Valentina Corino, digitaL tEchnologies for imaGing and sensOrs (LEGO) Lab, CCM
Address: via Parea 4, 20138 Milan, Italy
E-mail: valentina.corino@cardiologicomonzino.it

STATISTICAL ANALYSIS
Contact Name: Dr. Alice Bonomi, Biostatistics Unit, CCM
Address: via Parea 4, 20138 Milan, Italy
Tel: +39 02 58002828
E-mail: alice.bonomi@cardiologicomonzino.it

IMP MANAGMENT
Contact Name: Euromed Pharma Services S.r.l.
Address: Via Abruzzi,20056 Grezzago, Milan, Italy
Tel: +39 02 950698871
E-mail: andrea.mazzini@euromed-pharma.com

INVESTIGATIONAL SITES
Full list of investigational sites will be kept in the Trial Master File. Updated versions, if any, will be filed chronologically. Copies will be provided to the sites.

List of Abbrevations

AE	Adverse Event
ACM	Arrhythmogenic Cardiomyopathy
ADR	Adverse Drug Reaction
AORN	Azienda Ospedaliera di Rilievo Nazionale
AOU	Azienda Ospedaliera Universitaria
ASR	Annual Safety Report
CCM	Centro Cardiologico Monzino, Istituto di Ricovero e Cura a Carattere Scientifico, S.p.A.
CMR	Cardiac Magnetic Resonance
CRA	Clinical Research Associate
CRF	Case Report Form
CV	Cardiovascular
CVBF	Consorzio per Valutazioni Biologiche e Farmacologiche
DRE	Disease Related Events
ECG	Electrocardiogram
ECHO	Echocardiography
eCRF	Electronic Case Report Form
ESC	European Society of Cardiology
GMP	Good Manufacturing Practice
HADS	Hospital Anxiety and Depression Scale
HIPAA	Health Insurance Portability and Accountability Act
ICD	Implantable cardioverter defibrillator
ICF	Informed Consent Form
ICH	International Conference on Harmonization
IEC	Independent Ethics Committee
ILR	Implantable loop recorder
IES-R	Impact of Event Scale-Revised
IMP	Investigational Medicinal Product
IQR	Interquartile range
IRB	Institutional Review Board
ISF	Investigator Site File
ITT	Intent to Treat
LDL	Low density lipoprotein
NYHA	New York Heart Association
oxLDL	Oxidized low density lipoproteins
PKP2	Plakophilin2
PVC	Premature ventricular contraction
RV	Right ventricle
SAE	Serious Adverse Event
SD	Standard deviation
SEARCH	Statin Effect on ARrhythmogenic CardiomyopatHy disease progression
SF-36	Short Form Health Survey 36
SMB	Safety Monitoring Board
SmPC	Summary of Product Characteristic
SoA	Schedule of Activities
SRF	Safety Report Form

SUSAR	Suspected Unexpected Serious Adverse Reaction
T	Time
VA	Ventricular Arrhythmias
VF	Ventricular Fibrillation
VT	Ventricular Tachycardia
WOCBP	woman of childbearing potential

Protocol Amendment Summary of Changes Table

DOCUMENT HISTORY	
**Document**	**Date**
Amendment 1 Version 4.0	*[30-01-2025]*
Version 3.0	*[01-10-2024]*
Version 2.0	*[05-09-2024]*
Version 1.0	*[25-06-2024]*

TABLE OF CONTENTS

Sommario

1. Synopsis 12

2. Schedule of Activities (SoA) 16

3. Introduction 18

3.1. Study Rationale 18

3.2. Background 18

3.3. Benefit/Risk Assessment 19

4. Objectives and Endpoints 21

5. Study Design 22

5.1. Overall Design 22

5.2. Participant and Study Completion 23

5.3. Study Time table 23

5.4. End of Study Definition 23

5.5. Rationale for Study Design 23

5.6. Justification for Dose 25

6. Study Population 26

6.1. Inclusion Criteria 26

6.2. Exclusion Criteria 26

6.3. Lifestyle Restrictions 27

6.3.1. Meals and Dietary Restrictions 27

6.3.2. Caffeine, Alcohol, and Tobacco 27

6.3.3. Activity 27

6.4. Screen Failures 27

6.5. Assignment of patient number 28

7. Treatments 29

7.1. Treatments Administered 29

7.2. Dose Modification/ Adjustment 29

7.3. Method of Treatment Assignment 29

7.4. Blinding 29

7.5. Preparation/Handling/Storage/Accountability 30

7.5.1. Manufacturing, Packaging and Labelling of IMP 31

7.5.2. Supply, Storage and Handling of IMP 31

7.5.3. Accountability of the IMP 31

7.5.4. Treatment Compliance 32

7.6. Concomitant Therapy/Auxiliary medicinal product 32

7.6.1. Reporting of prior and concomitant medications 32

7.6.2. Restriction on allowed prior and concomitant medications 32

7.7. Treatment after the End of the Study 32

8. Study Assessments and Procedures 33

8.1. Enrolment, Screening and randomization 33
8.1.1. Enrolment 33
8.1.2. Screening 33
8.1.3. Randomisation 33
8.1.4. Visits 33
8.2. Discontinuation/Withdrawal Criteria 35
8.2.1. Discontinuation of Study Treatment 35
8.2.2. Temporary Discontinuation of the Study Treatment 36
8.2.3. Withdrawal from the Study 36
8.2.4. Lost to Follow-Up 36
8.3. Adverse Events 37
8.3.1. Definitions 37
8.3.1.1. Adverse Event 37
8.3.1.2. Adverse Reaction 37
8.3.1.3. Serious Adverse Event 37
8.3.1.4. Serious Adverse Reaction 37
8.3.1.5. Unexpected Adverse Drug Reaction 38
8.3.2. Recording of adverse events 38
8.3.2.1. Seriousness of AE 38
8.3.2.2. Relationship of AE to the IMP 38
8.3.2.3. Severity of AEs 39
8.3.3. Serious Adverse Events Reporting 39
8.3.3.1. Notification process 39
8.3.3.2 Regulatory Reporting Requirements for SAEs 40
8.3.4. Cardiovascular and Death Events 40
8.3.5. Disease-Related Events and/or Disease-Related Outcomes Not Qualifying as AEs or SAEs 40
8.3.6. Pregnancy 41
8.3.7. Code breaking 41
8.3.7.1. Emergency procedures 41
8.3.7.2. Pharmacovigilance purposes 41
8.4. Treatment of Overdose 42
8.5. Safety Assessments 42
8.5.1. Physical Examinations 42
8.5.2. Vital Signs 43
8.5.3. Electrocardiograms 43
8.5.4. Clinical Safety Laboratory Assessments 43
8.5.5. Genetics 43
8.5.6. Biomarkers 43
8.5.7. Metabolomic Research 43
9. Statistical Considerations 45
9.1. Sample Size Determination 45
9.2. Populations for Analyses 45
9.3. Statistical Analyses 45
9.1.1 Demographic and baseline characteristics 46

9.1.2 Analysis of efficacy variables 46
9.1.3 Analysis of safety variables 46
9.1.4 Missing data 46

10. Ethical Considerations 48
10.1. Independent Ethics Committee (IEC) 48
10.2. Informed consent 48
10.3. Insurance 49
10.4. Confidentiality and protection of Personal Data 49
10.5. Publication Policy 50

11. Data Handling and Record Keeping 51
11.1. Case Report Form 51
11.2. Data management 51

12. Study Management 52
12.1. Monitoring 52
12.2. Audit and Inspection 52
12.3. Protocol Deviations/amendments 52

13. References 53

14. Annexes List 56
Annex I: CTFG - Recommendations related to contraception and pregnancy testing in clinical trials 56
Annex II: Packaging and labelling details 56
Annex III: Drug Accountability 56
Annex IV: Psicological questionnaries (Annex IVa: HADS; Annex IVb: IES-R; Annex IVc: SF36) 56
Annex V: Clinical Laboratory Tests 56

1. Synopsis

Protocol Title: Statin Effect on ARrhythmogenic CardiomyopatHy disease progression

Short Title: SEARCH

Rationale: Retrospective studies in Arrhythmogenic Cardiomyopathy (ACM) patients of CCM showed that oxidized low density lipoproteins (oxLDL) plasma levels were associated with severe ACM phenotypes, as increased cardiac lipid accumulation, cardiac dysfunction, and arrhythmic burden. Preclinical studies in an ACM mouse showed that Atorvastatin treatment reduced oxLDL levels and prevented ACM phenotype development [1]. Based on these findings, we hypothesize that the use of Atorvastatin in ACM patients may delay disease progression toward heart failure and reduce electrical instability.

Objectives and Endpoints

Objectives	Endpoints	Measure of the outcome
**Primary**<br>Efficacy of Atorvastatin in avoiding functional RV deterioration	Deterioration from baseline of RV free wall longitudinal strain measured by ECHO.	Variation of strain RV free wall longitudinal strain measured by ECHO at T18 respect to T0 (%)
**Secondary**<br>**a)** Efficacy of Atorvastatin in avoiding electric, morphological and biomarkers deterioration;<br><br>**b)** safety of Atorvastatin treatment	**a)** Deterioration from baseline of i) arrhythmia burden (PVC, non-sustained and sustained VA, VF, appropriate ICD shocks); ii) other morphological parameters (ventricular volumes, function, both at ECHO and CMR); iii) ECG parameters; iv) blood parameters;<br><br>**b)** monitoring of AE and patient's well-being.	**a)**<br>i) Deterioration from baseline of the frequency of premature ventricular contractions, non-sustained and sustained ventricular arrhythmias, episodes of ventricular fibrillation, appropriate defibrillator shocks (measured by Holter, defibrillator and loop recorder interrogation at T18).<br>ii) Variation from baseline of morphological parameters, such as ventricular volumes (ml), parietal thicknesses (mm), fibrotic replacement characteristics (mm^2), cardiac functional parameters as ejection fraction (%) or Fractional shortening (%), heart movements (number and extension of bulging and wall motion abnormalities) measured at ECHO and CMR at T18.<br>iii) Variation from baseline of the electrocardiogram parameters (ms or voltage) at T18.<br>iv) Variation from baseline of biomarkers at T18 (concentrations, Units).<br>**b)**<br>i) Number and severity of adverse events (blood safety parameters variation and number and severity of patient-reported adverse effects) till T18.<br>ii) Variation in the score of the psychological questionnaires at T18 respect to baseline.
**Tertiary/Exploratory**<br>**a)** Validation of prediction models of ACM progression;<br><br>**b)** prediction of differential response to Atorvastatin treatment.	**a)** Testing the prediction models on the prospectively collected clinical, biological, genetic, and radiomics data of the placebo cohort;<br><br>**b)** testing the association of prospectively collected clinical, biological, genetic, and radiomics data of the Atorvastatin cohort with outcome.	**a)** Verification in terms of sensitivity, specificity, overall predictive performance of the correspondence between the predicted outcome (by different prediction models) and the actual variation at T18 of the clinical, biological, genetic, and radiomics variables of the placebo cohort;<br><br>**b)** association coefficients of the baseline clinical, biological, genetic, and radiomics variable of the Atorvastatin cohort with endpoints outcomes.

Overall Design:
This is a multicenter, prospective, randomized, clinical study: Atorvastatin 80mg/day or placebo will be administered for 18 months to 102 ACM patients, and the endpoints will be tested.
Atorvastatin has the potential to target ACM progression, thus reducing disease burden and related healthcare costs. The repurposing strategy drastically cuts the timing for its effective and economical use in ACM, respect to new drug development.
The clinical/biological data collected in the trial will also be used to validate a disease progression prediction model, and a to identify markers associated to Atorva differential response, with the use of innovative AI based tools.

Number of Participants:
Approximately 110 participants will be screened to enroll 102 patients. The final number of needed evaluable participants is 88 (taking into account the drop-outs) for an estimated total of 44 evaluable participants per treatment group.

Inclusion Criteria:
- Diagnosis of ACM based on the 2010 Task Force criteria or Padua Criteria [2,3]
- Age ≥ 18 years
- Signed the informed consent.

Exclusion Criteria:
- Known hypersensitivity to atorvastatin or any of the excipients
- Moderate or severe liver disease (persistent elevation of transaminases more than 3 times the upper limit of the normal laboratory reference range)
- Muscle disease (CK levels significantly elevated more than 3 times the upper limit of the normal laboratory reference range)
- Left ventricular ejection fraction <35%
- Congestive heart failure defined by the New York Heart Association (NYHA) as class III or IV.
- Known cardiomyopathy of other origin: post ischemic, hypertrophic, idiopathic dilated, restrictive; known moderate-to-severe mitral and/or aortic valvulopathy; pulmonary hypertension; congenital cardiac abnormalities
- Heart transplantation
- Estimated life expectancy of less than 2 years
- Any other medical condition that, in the judgment of the investigator, places the patient at risk or makes the patient unreliable or limits the patient's ability to complete the study
- Potent CYP3A4 modifiers such as Erythromycin, Clarithromycin Azole antifungals (e.g. itraconazole, posaconazole, voriconazole) Protease inhibitors (e.g. ritonavir, telaprevir, boceprevir), Gemfibrozil, Ciclosporin, Danazol
- Fusidic acid (drug for bacterial infections)
- Hepatitis C antivirals as telaprevir, boceprevir, glecaprevir/pibrentasvir and ledipasvir/sofosbuvir combination
- Any other lipid lowering drugs such as Statins (Atorvastatin, Fluvastatin, Lovastatin, Pravastatin, Rosuvastatin, Simvastatin) Cholesterol absorption inhibitors (Ezetimibe), Bile acid sequestrants (Cholestyramine, Colestipol), PCSK9 inhibitors (Alirocumab, Evolucumab), Adenosine triphosphate-citrate lyase inhibitors (Bempedoic acid), Fibrates (Gemfibrozil, Fenofibrate, Bezafibrate), Omega-3 fatty acid ethyl esters, and patients who have cholesterol levels that, according to guidelines [4], require the use of such drugs.
- Drugs primary indicated as antioxidants (N-acetyl-cysteine)

- Enrollment in another clinical trial or past clinical trial in which an investigational drug was administered within 30 days of Visit 1 or within the 5 half-lives of the investigational drug, whichever is longer.
- Pregnant or lactating women
- Women of childbearing age who are not using adequate contraception that complies with local regulations on methods of contraception for clinical trial participants (see section 6.1 and 6.2).
- Known dependency on alcohol – drug abuse.

Treatment Groups and Duration:

I) Atorvastatin 80 mg, 1 tablet/die, for 18 months
II) Placebo, 1 tablet/die, for 18 months

2. Schedule of Activities (SoA)

**Procedure**	**T0 Visit**		**Treatment Period 18 months**						**Follow-up Phone call, end of the study**
			**T2 Visit, Safety check**	**T4 Visit, Safety check**	**T9 Visit, data collection**	**T12 Phone call, safety check**	**T15 Phone call, safety check**	**T18 Visit, data collection**	
	Day 0 **Screening**	Day 0 **Randomization and data collection**	2nd month +/- 1 month	4th month +/- 1 month	9th month +/- 1 month	12th month +/- 1 month	15th month +/- 1 month	18th month +/- 1 month	19th month +/- 1 month
Informed consent	X								
Inclusion and exclusion criteria	X								
Randomization		X							
IMP supply		1 kit	2 kits		3 kits				
Demography	X								
Full physical examination		X	X	X	X			X	
Medical history (includes substance usage [and Family history of premature CV disease])		X							
Past and current medical conditions	X								
Pregnancy test (WOCBP only)	X		X	X	X			X	
Blood withdrawal for hepatic and muscle functionality	X		X	X	X			X	
12-lead ECG		X			X			X	
ECHO		X			X			X	
CMR		X**$^{\#}$						X$^{\#}$	
Holter ECG		X			X			X	
ICD/ILR interrogation		X***			X***			X***	

Procedure	T0 Visit		Treatment Period 18 months						Follow-up Phone call, end of the study
			**T2 Visit, Safety check**	**T4 Visit, Safety check**	**T9 Visit, data collection**	**T12 Phone call, safety check**	**T15 Phone call, safety check**	**T18 Visit, data collection**	
Psychological questionnaires		X			X			X	
Blood withdrawal for genetics		X*							
Blood withdrawal for ACM biomarkers		X			X			X	
Patient-reported outcome collection			X	X	X	X	X	X	X
Study treatment adherence check			X	X	X	X	X	X	
AE review			X	X	X	X	X	X	X
SAE review			X	X	X	X	X	X	X

* only if not preformed previously
** unless CMR performed within 3 months prior to study enrolment
# unless patient has contraindication for CMR
*** if present

3. Introduction

Arrhythmogenic Cardiomyopathy (ACM) is a rare genetic disease characterized by ventricular arrhythmias, increased sudden death risk, and progressive myocardial fibro-adipose replacement leading to heart failure [5]. It is identified by the following OMIM numbers: #609040, #607450, #610193, #610476, #611528, #107970, #604400, #615616, #618920, %602086, %602087, %604401 and forms with unknown genetic origin.
ACM affects ~90,000 people in Europe (prevalence of 1:5000 [6]), mainly young and athletes [7], with a dramatic impact on health and quality of life. Up to 25% of sudden deaths in age <30 years are caused by ACM [8-10]. The affected patients are at risk of cardiac sudden death and heart failure. Thus, they are subjected to a long-life follow-up to monitor symptom worsening and disease progression for cardiac morphological and/or functional impairments, and to optimize the treatment [11].
Current medications include beta blockers, antiarrhythmics, and heart failure drugs [12]. Implantable cardioverter defibrillator is life-saving, despite the morbidity linked to complications and inappropriate shocks. Other invasive procedures are available, such as ablation and end-stage heart transplantation. However, none of these approaches effectively address the underlying pathology. In particular, no specific therapies to prevent the fibro-adipose substitution are available.

3.1. Study Rationale

The present SEARCH TRIAL relies on solid preliminary *in vitro* and *in vivo* data suggesting novel pharmacologically targetable ACM pathogenic mechanisms. Retrospective studies in ACM patients of Centro Cardiologico Monzino IRCCS showed that oxidized low density lipoproteins (oxLDL) plasma levels were associated with severe ACM phenotypes, as increased cardiac lipid accumulation, cardiac dysfunction, and arrhythmic burden. Preclinical studies in an ACM mouse showed that Atorvastatin treatment reduced oxLDL levels and prevented ACM phenotype development [1]. Based on findings of the Centro Cardiologico Monzino IRCCS, we propose that the use of statins in patients suffering from ACM may have beneficial effects in reducing the progression of the pathology and avoiding the most severe consequences of the disease. Indeed, beyond the described canonical lipid-lowering effects, atorvastatin has known pleiotropic effects, as anti-oxidant, anti-inflammatory and anti-arrhythmic, leading, in ACM patients, to lowering of the triggering factor oxLDL, reduction of the inflammatory mediated disease bursts, and moderation of the arrhythmia burden.

3.2. Background

CCM *in vitro* and *in vivo* data laid the basis to propose a novel pharmacological treatment for ACM patients [9]. Briefly, to explain incomplete penetrance and phenotypic variability of ACM, we have shown that an increase in circulating oxidized low-density lipoproteins (oxLDL) is associated with more severe phenotypes (increased cardiac lipid accumulation, RV dysfunction and arrhythmic burden).
We confirmed our hypothesis in disease-relevant models (primary cardiac mesenchymal stromal cells and patient specific induced pluripotent stem cell-derived cardiomyocytes; heterozygous knock-out mouse model for plakophilin 2 (*PKP2*) identifying the molecular mechanism involved [1].
Preclinical studies in the mouse model showed that treatment with atorvastatin, reducing levels of oxLDL, prevents the severe manifestation of the mouse model disease phenotypes (increased cardiac lipid accumulation, cardiac dysfunction, electrocardiographic manifestations) [1].

3.3. Benefit/Risk Assessment

If the hypothesis proves to be correct, we expect to slowing down disease progression, both from the tissue and the electrical points of view, and consequently an improvement in symptoms and a reduction in arrhythmic risk.

The proposed therapy is expected to bring significant clinical benefit compared to existing therapies in Arrhythmogenic Cardiomyopathy context, none of which targets the fibro-adipogenic disease progression. The use of statins in patients suffering from ACM may have beneficial effects in reducing the progression of the pathology and avoiding the most severe consequences of the disease. Indeed, beyond the described canonical lipid-lowering effects, atorvastatin has known pleiotropic effects, as anti-oxidant, anti-inflammatory and anti-arrhythmic, leading, in ACM patients, to lowering of the triggering factor oxLDL, reduction of the inflammatory mediated disease bursts, and moderation of the arrhythmia burden.

The expected results will be beneficial for the health system, reducing costs and efforts for the management of ACM patients.

In addition, the patient in the study will be guaranteed close follow-up and more in-depth care than during routine visits.

However, it is possible that a subgroup of patients taking Atorvastatin may experience side effects.

In the database of placebo-controlled atorvastatin clinical trials involving 16,066 patients (8755 Atorvastatin vs. 7311 placebo) treated for an average period of 53 weeks, 5.2 per cent of patients on atorvastatin discontinued the trial due to adverse reactions, compared to 4.0 per cent of placebo-treated patients.

Below is the profile of atorvastatin adverse reactions based on data from clinical studies and extensive post-marketing experience. The frequencies of the estimated reactions are classified according to the following convention: common (≥1/100, <1/10); uncommon (≥1/1,000, <1/100); rare (≥1/10,000, <1/1,000); very rare (<1/10,000); frequency unknown (cannot be defined based on available data).

Infections and infestations
Common: nasopharyngitis.
Haemolymphopoietic system disorders
Rare: thrombocytopenia
Immune system disorders
Common: allergic reactions
Very rare: anaphylaxis
Metabolism and nutrition disorders
Common: hyperglycaemia
Uncommon: hypoglycaemia, weight gain, anorexia
Psychiatric disorders
Uncommon: nightmares, insomnia
Nervous system disorders
Common: headache
Uncommon: dizziness, paresthesia, hypoesthesia, dysgeusia, amnesia
Rare: peripheral neuropathy
Frequency not known: myasthenia gravis
Eye disorders
Uncommon: blurred vision
Rare: visual disturbance
Frequency not known: ocular myasthenia
Ear and labyrinth disorders
Uncommon: tinnitus

Very rare: hearing loss
Respiratory, thoracic and mediastinal disorders
Common: pharyngolaryngeal pain, epistaxis
Gastrointestinal disorders
Common: constipation, flatulence, dyspepsia, nausea, diarrhoea
Uncommon: vomiting, upper and lower abdominal pain, belching, pancreatitis
Hepatobiliary disorders
Uncommon: hepatitis
Rare: cholestasis
Very rare: liver failure
Skin and subcutaneous tissue disorders
Uncommon: urticaria, rash, itching, alopecia
Rare: angioneurotic oedema, bullous dermatitis, including erythema multiforme, Stevens-Johnson syndrome and toxic epidermal necrolysis
Musculoskeletal system and connective tissue disorders
Common: myalgia, arthralgia, pain in extremities, muscle spasms, joint swelling, back pain
Uncommon: neck pain, muscle fatigue
Rare: myopathy, myositis, rhabdomyolysis, muscle rupture, tendinopathy, sometimes complicated by rupture
Very rare: Lupus-like syndrome
More detailed information about the known and expected benefits and risks and reasonably expected adverse events of Atorvastatin may be found in the Summary of Product Characteristic (SmPC).

Overall, the anticipated benefits to the subjects and to public health justify the foreseeable risks and inconveniences and compliance with this condition is constantly monitored

4. Objectives and Endpoints

Objectives	Endpoints	Measure of the outcome
**Primary**<br>Efficacy of Atorvastatin in avoiding functional RV deterioration	Deterioration from baseline of RV free wall longitudinal strain measured by ECHO.	Variation of strain RV free wall longitudinal strain measured by ECHO at T18 respect to T0 (%)
**Secondary**<br>**a)** Efficacy of Atorvastatin in avoiding electric, morphological and biomarkers deterioration;<br><br>**b)** safety of Atorvastatin treatment	**a)** Deterioration from baseline of i) arrhythmia burden (PVC, non-sustained and sustained VA, VF, appropriate ICD shocks); ii) other morphological parameters (ventricular volumes, function, both at ECHO and CMR); iii) ECG parameters; iv) blood parameters;<br><br>**b)** monitoring of AE and patient's well-being.	**a)**<br>i) Deterioration from baseline of the frequency of premature ventricular contractions, non-sustained and sustained ventricular arrhythmias, episodes of ventricular fibrillation, appropriate defibrillator shocks (measured by Holter, defibrillator and loop recorder interrogation at T18).<br>ii) Variation from baseline of morphological parameters, such as ventricular volumes (ml), parietal thicknesses (mm), fibrotic replacement characteristics (mm^2), cardiac functional parameters as ejection fraction (%) or Fractional shortening (%), heart movements (number and extension of bulging and wall motion abnormalities) measured at ECHO and CMR at T18.<br>iii) Variation from baseline of the electrocardiogram parameters (ms or voltage) at T18.<br>iv) Variation from baseline of biomarkers at T18 (concentrations, Units).<br>**b)**<br>i) Number and severity of adverse events (blood safety parameters variation and number and severity of patient-reported adverse effects) till T18.<br>ii) Variation in the score of the psychological questionnaires at T18 respect to baseline.
**Tertiary/Exploratory**<br>**a)** Validation of prediction models of ACM progression;<br><br>**b)** prediction of differential response to Atorvastatin treatment.	**a)** Testing the prediction models on the prospectively collected clinical, biological, genetic, and radiomics data of the placebo cohort;<br><br>**b)** testing the association of prospectively collected clinical, biological, genetic, and radiomics data of the Atorvastatin cohort with outcome.	**a)** Verification in terms of sensitivity, specificity, overall predictive performance of the correspondence between the predicted outcome (by different prediction models) and the actual variation at T18 of the clinical, biological, genetic, and radiomics variables of the placebo cohort;<br><br>**b)** association coefficients of the baseline clinical, biological, genetic, and radiomics variable of the Atorvastatin cohort with endpoints outcomes.

5. Study Design

5.1. Overall Design

This is a multicenter, prospective, randomized, double-blind, placebo-controlled Phase II clinical trial.

The duration of the study for a participant will include:

- patients' enrolment: patients will be enrolled, after written informed consent obtainment, if they meet inclusion criteria and no exclusion criteria are verified.
- randomization: once successfully screened, enrolled participants will receive IMP (either Atorvastatin or placebo)
- treatment period: the patient will take the IMP daily for 18 months. At the beginning (T0 visit) data collection will be performed. During this period the patient will be seen at the second and fourth month for a safety check (T2 and T4 visit), to control for hidden adverse reactions, at month 9 (T9 visit) and at month 18 (T18 visit) for data collection and safety check. Meanwhile, 2 telephone call, at T12 and T15 will be performed to collect patient reported status. In addition, unscheduled visits, for safety reasons are welcome.
- follow-up period: a safety follow-up telephonic check will be performed approximately 1 months after the last dose of IMP (T19).

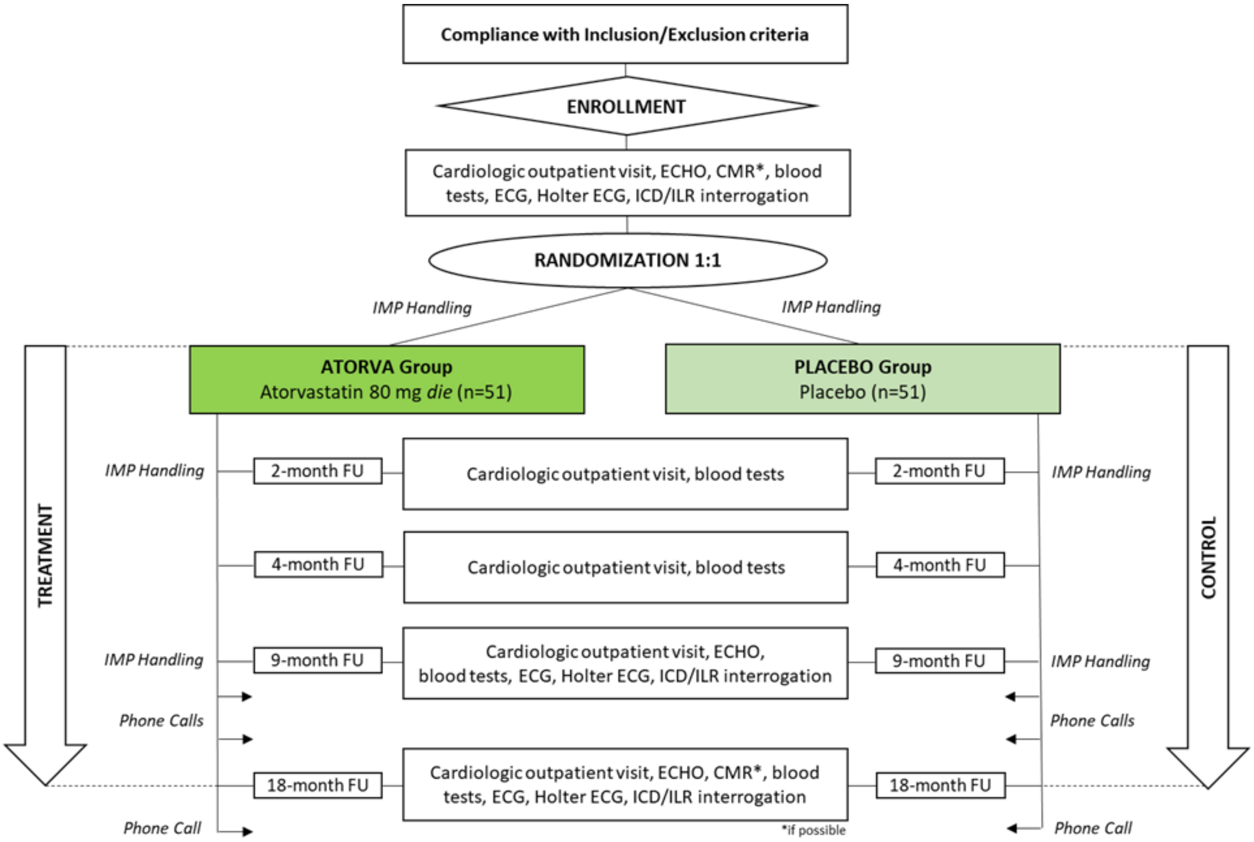

5.2. Participant and Study Completion

Approximately 110 participants will be screened to achieve 102 randomly assigned to study treatment and 88 evaluable participants for an estimated total of 44 evaluable participants per treatment group.

5.3. Study Time table

Planned starting date (first-patient-in): October, 2024
Projected completion of patient accrual (last-patient-in): March 2025
Projected study end date (last-patient-last-visit): August 2026
Each patient participation is expected to be 19 months from T0 screening visit to T19 follow-up telephone call. This period includes 18 months of treatment and 1 month follow-up.

5.4. End of Study Definition

A participant is considered to have completed the study if he/she has completed all phases of the study including the last visit at T18 and having received the telephone call at T19.
The end of the study is defined as the date of the last telephone call at T19 of the last participant in the study.

5.5. Rationale for Study Design

Study design. We decided to propose a phase II randomized trial since Atorvastatin is a known, safe and broadly used drug. The safety studies on healthy controls (phase I trials) have been already performed. We will use atorvastatin in a posology as per technical data sheet.
The use of placebo is proposed in order to control confounding factors (e.g. patient and physician suggestions, spontaneous fluctuation of the disease and its symptoms) that may affect the reading of the research results. In particular, the use of the placebo could help in the actual estimation of the side effects of Atorvastatin and the perception of the symptoms of the disease, and of patients's well-being, thus being useful in the secondary endpoint.
Choice of the IMP. Atorvastatin was chosen among statins as has been tested both *in vitro* and *in vivo*. Among the statins it is one of those with a high antioxidant effect [13]. The choice of the brand (Atorvastatina TEVA) is due to the fact that the tablet is relatively simple and smooth, with no color and no writing, therefore easy to reproduce as a placebo tablet. In addition, it is available and easily accessible on the market in Italy.
Choice of primary endpoint. Only few interventional clinical studies for therapy validation have been performed or are ongoing on ACM patients. Some are Phase1, therefore the primary endpoint is safety and tolerability (NCT06109181, NCT06228924, NCT05885412). The ones that test efficacy of therapeutics are the following:
- NCT03685149: “Pilot Randomized Trial With Flecainide in ARVC Patients” to test the efficacy of Flecainide in the reduction of number of ventricular ectopic beats.
- NCT03593317: “Blockade of the Renin-angiotensin-aldosterone System in Patients With ARVD (BRAVE)” to test the efficacy of spironolactone on ventricular myocardial function and remodeling (measured by right ventricle longitudinal strain and right ventricle infundibulum diameter at ECHO) and arrhythmia burden (number of ventricular extrasystoles).
-NCT06174220. Targeted Therapy With Glycogen Synthase Kinase-3 Inhibition for Arrhythmogenic Cardiomyopathy to test ventricular function (change in ventricular strain at ECHO) and PVC burden.
-NCT06275893: IC14 “(Atibuclimab) in Arrhythmogenic Cardiomyopathy” to determine whether IC14 treatment reduces markers of inflammation and disease biomarkers in ACM patients treated with IC14.

-NCT05524077: “Catheter Ablation Versus Anti-arrhythmic Drugs for Ventricular Tachycardia (CAAD-VT)” to test a composite endpoint of Recurrent VT or VT storm or death following the different treatments.

Common primary endpoints are RV function or arrhythmia burden. Those trials assessing RV function use RV strain as outcome.

Accordingly, we chose the primary endpoint outcome to be a clinically relevant measurable parameter. We chose RV longitudinal strains for the following reasons:

- CCM *in vitro* results point to an action of atorvastatin in counteracting the adipogenic differentiation of cardiac cells leading to a moderation of morphological/functional remodeling of the cardiac substrate (of which strain is a measure [14]).
- CCM *in vivo* results showed a significant involvement of the right ventricle respect to the left ventricle.
- RV free wall longitudinal strain reflects the deformation of the RV in the longitudinal direction. In comparison with RV ejection fraction, it is earlier in the detection of systolic disfunction; moreover, it is linked to prognosis in many clinical conditions.
- Free wall longitudinal strain of the RV worsens over the years in ACM patients with little variability, as proven by the CCM cohort retrospective data (n=20 patients with double ECHO): 12.8% (2.4 points of strain) reduction in 18 months with a standard deviation of 4.5%.
- A similar difference of RV strain has recently been confirmed by other group publications [15],[16]. In addition, a similar standard deviation was measured in other cohorts [14].

ECHO parameters are measurable in every patient, while CMR is feasible only if the patient is not carrying an ICD or if the ICD is compatible with CMR. In the last case it suffers of some artifacts.

Secondary endpoints. Secondary endpoints will include other information regarding disease progression. Importantly, arrhythmia burden will be monitored, and it is expected to decrease, as reported for other non-ischemic populations in treatment with statins [17]. Most known clinical markers and biomarkers will be assessed, as well as the novel biomarker oxLDL [1]. In addition, it is important to evaluate safety of Atorvastatin 80 mg administration for the novel indication (ACM patients).

Exclusion criteria. They have been chosen to limit as much as possible the side effects caused by the treatment.

Length of the treatment. The choice of the 18-months treatment is due to the fact that ACM is a progressive disease but in the majority of patients, slowly progressive. A retrospective analysis of our cohort showed a quantifiable mean progression, as RV free wall longitudinal strain in 18 months. Therefore, this is the minimum term in which we could detect a favorable effect of Atorvastatin treatment.

Power analysis. We calculated the trial sample size basing on the clinical data of CCM patient cohort, with double Echo, because little data on disease progression, above all functional cardiac data, are available in the literature. However, a similar sample size has been estimated, based on RV strain, in other, currently recruiting, pharmacological trials in ACM patients (NCT03593317 and NCT06174220), who recruit 120 patients and measure the outcome at 1 year or 6 months respectively.

In calculating the sample size, we had to keep into account that ACM is a rare disease, therefore we had to discard all the endpoints that we tested which gave as result an unreachable sample size. A reachable criterion would have been the quantity of PVC in the 24 hours. However, we believe that the effect of atorvastatin is only indirectly likely to affect arrhythmic burden (since it was demonstrated to impact the RV function, primarily). Besides, it is very variable and it would have needed a larger cohort.

In addition, we have been conservative in considering 15% possible drop-out, since we are aware that it is more likely that an adverse event or patient withdrawal happens in the context of a long treatment.

Patient recruitment. Patients will be recruited throughout different centres in Italy. The involvement of several centres is essential to guarantee the recruitment of many patients in the context of a disease with a prevalence of 1:5000, of which the majority are male patients [18] and athletes are frequent [19].The inhomogeneity of the different cohorts will, from one part, account for a higher variability of the results, from the other will guarantee real word data, and applicability of the results of the trial to a broader ACM population.

Safety and compliance. Patients will be monitored approximately every 3 months either by an outpatient visit or through a telephone call, to screen adverse events, serious adverse events and suspected unexpected adverse events, and to check on therapy compliance. For safety needs, patients can be subjected to unscheduled visits.

5.6. Justification for Dose

The dose of the drug has been selected after an in-depth evaluation of the literature. Previous clinical trial demonstrated the efficacy of 80 mg/die orally atorvastatin to reduce oxLDL plasma concentrations, modulate oxidative stress and inflammation, as desirable in ACM patients [20,21]. The same dose is demonstrated to be safe and with a similar incidence of adverse effects if compared to lower doses [22,23]. In addition, the median duration of 80 mg atorvastatin treatment in previous clinical trials is 22,56 months, indicating the safety of the treatment for 18 months as proposed in our trial.

6. Study Population

Patients affected by the rare disease ACM [24] of both sexes, with an age ≥18 years old, will be enrolled, after written informed consent obtainment, if they meet inclusion criteria and no exclusion criteria are verified.

6.1. Inclusion Criteria

To be eligible for inclusion into this study, each patient must fulfil all the following criteria:

Age

- Participant must be at least 18 years of age, at the time of signing the informed consent.

Type of Participant and Disease Characteristics

- Participants affected by cardiomyopathy as defined by task force criteria and Padua Criteria [2] [24]
- Participants signed the informed consent.

Sex

Male and female participants will be enrolled.
Male participants:

A male participant is invited to agree to use contraception during the treatment period and refrain from donating sperm during this period.

Female participants:

Female of childbearing age, as defined in Annex I, may be enrolled if they are not in a state of established pregnancy or lactation, and must in any case agree to undergo a pregnancy test (beta hCG) at each visit: T0 – screening, T2, T4, T9 and at the end of the Study (T18). Women of childbearing age who will be enrolled must use appropriate effective contraception that complies with local regulations on methods of contraception for clinical trial participants, in accordance with the recommendations of the document *“CTCG Clinical Trials Coordination Group-Recommendations on Contraception and Pregnancy Testing in Clinical Trials- Version 1.2 dated March 7, 2024*” attached below (Annex I).

6.2. Exclusion Criteria

Participants are excluded from the study if any of the following criteria apply:

Medical Conditions

- Known hypersensitivity to atorvastatin or any of the excipients
- Moderate or severe liver disease (persistent elevation of transaminases more than 3 times the upper limit of the normal laboratory reference range)
- Muscle disease (CK levels significantly elevated more than 3 times the upper limit of the normal laboratory reference range)
- Left ventricular ejection fraction <35%
- Congestive heart failure defined by the New York Heart Association (NYHA) as class III or IV.
- Known cardiomyopathy of other origin: post ischemic, hypertrophic, idiopathic dilated, restrictive; known moderate-to-severe mitral or aortic valvulopathy; pulmonary hypertension; congenital cardiac abnormalities
- Hypercholesterolemic patients that, according to guidelines [4], require the use of lipid lowering drugs.

- Heart transplantation
- Estimated life expectancy of less than 2 years
- Any other medical condition that, in the judgment of the investigator, places the patient at risk or makes the patient unreliable or limits the patient's ability to complete the study

Prior/Concomitant Therapy

- Potent CYP3A4 modifiers such as Erythromycin, Clarithromycin Azole antifungals (e.g. itraconazole, posaconazole, voriconazole) Protease inhibitors (e.g. ritonavir, telaprevir, boceprevir), Gemfibrozil, Ciclosporin, Danazol
- Fusidic acid (drug for bacterial infections)
- Hepatitis C antivirals as telaprevir, boceprevir, glecaprevir/pibrentasvir and ledipasvir/sofosbuvir combination
- Any other lipid lowering drugs such as Statins (Atorvastatin, Fluvastatin, Lovastatin, Pravastatin, Rosuvastatin, Simvastatin) Cholesterol absorption inhibitors (Ezetimibe), Bile acid sequestrants (Cholestyramine, Colestipol), PCSK9 inhibitors (Alirocumab, Evolucumab), Adenosine triphosphate-citrate lyase inhibitors (Bempedoic acid), Fibrates (Gemfibrozil, Fenofibrate, Bezafibrate), Omega-3 fatty acid ethyl esters
- Drugs primary indicated as antioxidants (N-acetyl-cysteine)

Prior/Concurrent Clinical Study Experience

- Enrollment in another clinical trial or past clinical trial in which an investigational drug was administered within 30 days of Visit 1 or within the 5 half-lives of the investigational drug, whichever is longer.

Other Exclusions

- Pregnant or lactating women
- Women of childbearing age who are not using adequate contraception that complies with local regulations on methods of contraception for clinical trial participants (see section 6.1).
- Known dependency on alcohol – drug abuse.

6.3. Lifestyle Restrictions

6.3.1. Meals and Dietary Restrictions

The concomitant intake of large quantities of grapefruit juice and atorvastatin is not recommended.

6.3.2. Caffeine, Alcohol, and Tobacco

Avoid consuming too much alcohol while taking this medicine.

6.3.3. Activity

Participants will abstain from strenuous exercise before each blood collection for clinical laboratory tests.

As advised in clinical practice ACM patients will abstain from intensive exercise or competitive sports [25].

6.4. Screen Failures

Screen failures are defined as participants who consent to participate in the clinical study but are not subsequently randomly assigned to study treatment. A minimal set of screen failure information is required to ensure transparent reporting of screen failure participants to meet the Consolidated Standards of Reporting Trials (CONSORT) publishing requirements and to respond to queries from regulatory authorities. Minimal information includes demography, screen failure details, eligibility criteria, and any serious adverse event (SAE).

Individuals who do not meet the criteria for participation in this study (screen failure) may be rescreened. Rescreened participants should be assigned the same participant number as for the initial screening.

6.5. Assignment of patient number

Patient number will be assigned in a sequential manner as a patient is found to be eligible for entry into the study and is enrolled. It will consist of the site number, and the patient number e.g. 102, where first digit represents site number, last two digits patient number.

If a patient is dropped from the study for any reason, the patient's number will not be reassigned. Should a patient be replaced, his/her number will not be re-allocated. A new patient will be enrolled and assigned the next number available.

7. Treatments

Study treatment is defined as any investigational treatment(s) and placebo, intended to be administered to a study participant according to the study protocol.

7.1. Treatments Administered

**Treatment**	Atorvastatin Teva Italia	Placebo
**Dosage formulation**	Film-coated tablets, white, shaped elliptical, and coated with smooth film. The dimensions of each tablet are approximately 18.8 mm x 10.3 mm.	Film-coated tablets, white, shaped elliptical, and coated with smooth film. The dimensions of each tablet are approximately 18.8 mm x 10.3 mm.
**Unit dose strength(s)**	80 mg of atorvastatin calcium	-
**Daily dosage level(s)**	80 mg	-
**Route of Administration**	Orally	Orally
**Dosing instructions:**	One tablet/die to take with or without food indifferently	One tablet/die to take with or without food indifferently
**Packaging and Labeling**	Study Treatment will be provided in a primary packaging consisting of Alu/Alu blisters of 10 tablets each, and a secondary packaging consisting of a box of 12 blisters. Each blister and each box will be labeled as required per country requirement. Each labeled box containing labeled blisters constitutes a KIT	Placebo will be provided in a primary packaging consisting of Alu/Alu blisters of 10 tablets each, and a secondary packaging consisting of a box of 12 blisters. Each blister and each box will be labeled as required per country requirement. Each labeled box containing labeled blisters constitutes a KIT
**Manufacturer**	Teva Italia S.r.l.	Laboratorio Farmacologico Milanese S.r.l.

7.2. Dose Modification/ Adjustment

No dose modification is foreseen.

7.3. Method of Treatment Assignment

All patients who sign informed consent and begin the study screening process will be tracked through a 3-digit identification code that will be assigned at hospital admission at each study site using the electronic Case Report Forms (eCRF). The 1st digit will indicate the study site and the 2nd and 3rd digit numbers the individual patient number. Patients will be randomized 1:1 to determine treatment allocation on top of the conventional therapy. The randomization list will be generated using SAS 9.4 statistical software (version 9.4, SAS Institute, Cary, NC, USA). The randomization scheme will be securely stored at the coordinator centre. Once randomization is completed and a treatment is assigned, crossover will be not permitted. Randomized patients who terminate their study participation for any reason, regardless of whether study intervention was taken/completed or not, will retain their randomization code.

7.4. Blinding

The proposed study will be a double-blind study in which investigators and patients are not aware of group assignment. In order to maintain this blind, a 3rd party (Euromed Pharma Services S.r.l.) will be responsible of blinding and distribution to the sites of the study treatment and will endeavor to ensure that there are no differences in time taken to provide to the pharmacies of each site the following randomization:

- Atorvastatin 80 mg and placebo tablets will be indistinguishable in appearance as to maintain the study blind. Also, labelling will not allow to recognize actual treatment.

During the trial, blinding will be broken by the Investigator for emergency purposes only, where knowledge of the blinded treatment could influence further patient care. In addition, CVBF pharmacovigilance will receive safety reports, as per regulatory requirements.
Study blind will be broken after database lock.
In addition, in order to minimize bias in outcome assessment, the following blinding measures will be taken:

a) Blinded CMR and ECHO core laboratory: CMR and ECHO will be interpreted by 2 expert observers blinded to individual patient treatment assignments in a dedicated core laboratory (at proponent site). The imaging core laboratory is certified by European Society of Cardiology (ESC).
b) Blinded outcome analysis: an independent professional statistical team will be involved to assess the pre-specified outcomes. The Statistical team will be blinded to individual patient treatment assignments (the analysis will be performed on group A vs. group B, with the exception of Aim3 analysis).

Due to the blinded design of the study breaking code procedures are envisaged. In particular, the blind may be broken if, in the opinion of the investigator, it is in the participant's best interest for the investigator to know the study treatment assignment. The sponsor must be notified before the blind is broken unless identification of the study treatment is required for a medical emergency in which the knowledge of the specific blinded study treatment will affect the immediate management of the participant's condition. The randomization code can be unblinded upon written request to CCM, which retain the assignment correspondence. The date and reason that the blind was broken must be recorded in the source documentation and CRF, as applicable.
In the event of a Quality Assurance audit, the auditor(s) will be allowed access to unblinded study treatment records at the site(s) to verify that randomization/dispensing has been done accurately.

7.5. Preparation/Handling/Storage/Accountability

The Investigational Medicinal Product (IMP) consists of Atorvastatin Teva Italia 80 mg film-coated tablets (an approved prescription medicine - class A, based on atorvastatin calcium salt, belonging to the therapeutic group Statin hypolipidemizers) as 1 tablet/die or placebo.
Atorvastatin will be purchased from commercial vendors, placebo will be produced, in accordance with the principles and guidelines of Good Manufacturing Practice (GMP) for Medicinal Products, in an authorized pharmaceutical manufacturer. Batch release certificate will be provided together with the IMPs.
Packaging and labelling of both Atorvastatin and Placebo will be performed by Euromed Pharma Services S.r.l. in accordance with the Regulation (EU) No 536/2014 (see Annex II) In particular, kits will be prepared in boxes, containing 12 blisters of 10 tablets each and labelled with the randomization codes. Each participating centre will be supplied with an adequate number of IMP. The IMP will be securely stored at room temperature.
This trial will use the Redcap CRF as electronic drug accountability system and pharmacy records (attached as Annex III). All shipping and receiving invoices indicating the type and quantity of the IMP and the date of shipment will be recorded at each site. Responsible investigators at each centre are required to maintain adequate records of the disposition of the IMP.
After the randomization, the Investigators will record into the CRF the kit number assigned to the patient and will dispense an adequate number of kits to comply with the study treatment until the following visit.
Before receiving the first kit of study treatment, patients will be instructed in using, handling, storing, and returning of IMP.
At each study visit the patient will return the empty batches to the site and those close to the expiry dates. The investigators will check the reconciliation between dispensed and returned IMP and will dispense it again. The accountability will be documented into the CRF and in the pharmacy record form at each study visit.

In addition, to monitor the patient's compliance to IMP about every 3 months will be performed a telephone call-visit. The treatment will last 18 months for each patient.

7.5.1. Manufacturing, Packaging and Labelling of IMP

The IMP Atorvastatin will be purchased from commercial authorized vendors, placebo will be produced, by an authorized pharmaceutical manufacturer.
Atorvastatin Teva Italia 80 mg film-coated tablets will be packaged and labelled Euromed Pharma Services S.r.l.
The study medication will be provided as kits consisting of a box containing 12 blisters of 10 tablets each.
All labels will be prepared to meet local regulatory requirements.

7.5.2. Supply, Storage and Handling of IMP

An appropriate number of packages to cover the first 9 months of the study will be initially sent to the site as soon as all essential documents and regulatory/ethics approvals have been obtained. IMP re-supply is planned around 8 months after the study initiation, for the second batch of IMP, in order to respect the IMP expiry date. Kits produced in the 2nd tranche will keep the same randomisation code as the first, so that each patient always has the same code.
The IMP must be kept at a room temperature.
A temperature probe will accompany the drug on shipment. Temperature range reached during shipment will be verified on receipt, so that potential stability concerns during shipment can be investigated and appropriate action taken.
Once received at the site, the Pharmacist (or designee) will check the package for accurate delivery and acknowledge receipt; any deviations from expected package content (inconsistency, damages) should be immediately reported to CCM and the use of the drug suspended until authorization for its continued use has been given by CCM.
The IMP must be stored in a secure location, at room temperature.
The IMP will be dispensed only by the Pharmacist (or authorized designee). The Investigator will ensure that study treatment is only administered by designated staff within the centre.

7.5.3. Accountability of the IMP

All supplies will be maintained under adequate security by the designated member of site staff, until they are dispensed to the patients.
When the IMP is received by the designated member of site staff, (s)he will check for accurate delivery and acknowledge receipt by signing and dating the documentation provided by Euromed Pharma Services S.r.l. on behalf of CCM and returning it to Euromed Pharma Services S.r.l. A copy will be retained for the Investigator/Pharmacy file.
The dispensing of the IMP will be reported in the medical record and collected in the eCRF and appropriate drug accountability forms and an accurate accounting will be available for verification by the CRA at each monitoring visit. Immediately before dispensing each Treatment kit, it will be registered on the CRF (randomization ID, kit number (e.g. 1 out of 6), dispensation date, batch number and expiry date).
Drug accountability records, attached as Annex III, includes:

1. the confirmation of receipt of the IMP,
2. the dispensing of the IMP to the patient,
3. the receipt of IMP returned from the patient,
4. the disposition (destruction) of unused product(s),
5. accounts of any IMP discrepancy.

They should include dates, quantities, batch numbers, expiration dates and any unique randomization code numbers assigned to the IMP and patients. Investigators should maintain records which document adequately that:

1. the patients were provided the doses specified by the protocol/amendment(s),
2. the IMP provided was fully reconciled at the site.

The CRA will review the drug accountability forms and CRF and check all IMP (both unused and used) prior to making arrangements for their disposal.

IMP which has been dispensed to a patient and returned unused will not be re-dispensed to a different patient. Unused IMP must remain in the Treatment Kit and must not be discarded or used for any purpose. Any remaining test material at the end of the trial will be returned to Euromed Pharma Services S.r.l., before the expiry date.

7.5.4. Treatment Compliance

Adherence will be checked during each visit and the patient's compliance to IMP about every 3 months will be performed a telephone call/visit.

IMP tablet count is the method to assess compliance to therapy.

7.6. Concomitant Therapy/Auxiliary medicinal product

No standard of care therapy is reported for ACM, therefore no background therapy is mandated. Patients will continue to take their background therapy for the disease under study according to patient needs and to the cardiologists' judgement.

7.6.1. Reporting of prior and concomitant medications

The date and time of concomitant medications administration as well as the name and dosage regimen of the rescue medication must be recorded.

7.6.2. Restriction on allowed prior and concomitant medications

Apart from medications not allowed by the exclusion criteria, which would exclude the patient access to the study, the recruited patients will continue current therapy, and will add the IMP on top of that.

7.7. Treatment after the End of the Study

Following the conclusion of the trial, study participants will continue to be treated in accordance with the clinical practice.

8. Study Assessments and Procedures

A schedule for the tests and evaluations to be conducted in this study is found in the schedule of activities.
For all measurements, the actual date and time of assessment, including date of sampling, will be recorded in the CRF. Where a time window is acceptable, this is clearly indicated in the following sections.

8.1. Enrolment, Screening and randomization

8.1.1. Enrolment

Patients' enrolment: patients will be enrolled, after written informed consent obtainment, if they meet inclusion criteria and no exclusion criteria are verified.

8.1.2. Screening

A screening form in the CRF will be completed for all patients who signed the Patient Informed Consent Document, regardless of subsequent randomization into the study. Patients will be identified by their age and sex; patient number if randomized or reasons for exclusion from the study will be recorded.

8.1.3. Randomisation

Compliance with inclusion/exclusion criteria will be finally verified through demographic, laboratory test results and clinical information.
Patients fulfilling all the inclusion criteria and none of the exclusion criteria will be randomized (randomization number assigned) by an Investigator (or designee).
Patients who do not meet the Inclusion Criteria or meet Exclusion Criteria will be considered screen failures (screen failure number assigned) and will not be allowed to be re-considered for inclusion into the study.

8.1.4. Visits

T0 Visit (Day 0): Screening

The Investigator will explain to the patient(s) the objectives of the study, benefits and risks for the patient and will answer all questions regarding the study.
Following the signature of the informed consent, the participant’s inclusion/exclusion criteria will be assessed.
This visit includes:

- Signature of informed consent
- Evaluation of Inclusion and exclusion criteria
- Demography
- Past and current medical conditions
- Pregnancy test (WOCBP only)
- Blood withdrawal for hepatic and muscle functionality

Randomization and data collection

This includes:
Randomization

- IMP supply (1 kit) and instructions for IMP handling
- Full physical examination
- Medical history (includes substance usage)
- 12-lead ECG
- ECHO

- CMR
- Holter ECG
- ICD/ILR interrogation
- Psychological questionnaires (i.e. Short Form Health Survey 36 (SF-36) [26] ; Hospital Anxiety and Depression Scale (HADS) [27]; Impact of Event Scale-Revised (IES-R)) [28] (see Annex IVa, Annex IVb and Annex IVc)
- Blood withdrawal for genetics, if needed
- Blood withdrawal for biomarkers

T2 Visit Safety check (2nd month +/- 1 month)
This visit includes:

- IMP supply **(2kits)**
- Full physical examination
- Pregnancy test (WOCBP only)
- Blood withdrawal for hepatic and muscle functionality
- Patient-reported outcome collection
- Study treatment adherence check
- AE review
- SAE review

T4 Visit Safety check (4nd month +/- 1 month)
This visit includes:

- Full physical examination
- Pregnancy test (WOCBP only)
- Blood withdrawal for hepatic and muscle functionality
- Patient-reported outcome collection
- Study treatment adherence check
- AE review
- SAE review

T9 Visit Data collection (9th month +/- 1 month)
This visit includes:

- IMP supply (3 kits)
- Full physical examination
- Pregnancy test (WOCBP only)
- Blood withdrawal for hepatic and muscle functionality
- 12-lead ECG
- ECHO
- Holter ECG
- ICD/ILR interrogation
- Psychological questionnaires (i.e. SF-36; HADS; IES-R)
- Blood withdrawal for biomarkers
- Patient-reported outcome collection
- Study treatment adherence check
- AE review
- SAE review

T12 Phone call, safety check (12th months +/- 1 month)
This visit includes:

- Patient-reported outcome collection
- Study treatment adherence check
- AE review
- SAE review

T15 Phone call, safety check (15th months +/- 1 month)
This visit includes:
- Patient-reported outcome collection
- Study treatment adherence check
- AE review
- SAE review

T18 Visit, data collection (18th month +/- 1 month)
This visit includes:
- Full physical examination
- Blood withdrawal for hepatic and muscle functionality
- Pregnancy test (WOCBP only)
- 12-lead ECG
- ECHO
- CMR
- Holter ECG
- ICD/ILR interrogation
- Psychological questionnaires (i.e. SF-36; HADS; IES-R)
- Blood withdrawal for biomarkers
- Patient-reported outcome collection
- Study treatment adherence check
- AE review
- SAE review

Follow-up Phone call, end of the study (19th month +/- 1 month)
This visit includes:
- Patient-reported outcome collection
- AE review
- SAE review

8.2. Discontinuation/Withdrawal Criteria

8.2.1. Discontinuation of Study Treatment

Definitive treatment discontinuation for individual subjects may occur due to undesirable effects related to the systemic use of oral IMP as follows: severe allergic reaction; serious skin conditions; serious muscle weakness, soreness, pain or breakage or change in color of the urine (rhabdomyolysis) (rare; ≥1/1,000); unusual bleeding or bruising, suggesting a liver disorder; Lupus-like illness syndrome (very rare; <1/10,000).
The SMB will evaluate further “stopping rules” or “discontinuation criteria” based on side effects registered during the trial.
All definitive treatment discontinuation reasons and dates should be recorded by the investigators in the appropriate CRF page when considered as confirmed.

The study will be stopped if any of the following are found:

i. a death possibly/probably related to the IMP;
ii. treatment discontinuation (> 15% patients) due to severe adverse effects (SAEs) related to the systemic use of oral IMP.

If the IMP administration is prematurely discontinued the primary reason for discontinuation must be recorded in the CRF.

See the SoA for data to be collected at the time of treatment discontinuation and follow-up and for any further evaluations that need to be completed.

8.2.2. Temporary Discontinuation of the Study Treatment

Temporary treatment discontinuation is allowed (i.e. failing to take medication for ≤ 15 consecutive days).

8.2.3. Withdrawal from the Study

Patients will be informed that they have the right to withdraw from the study at any time (withdrawal of consent), without prejudice to their medical care, and are not obliged to state their reasons. Patients who have withdrawn consent to the study or who for any cause have discontinued the study cannot be reincluded in it. Investigators may decide to discontinue the study for a patient for any clinical problem, or if other issues arise (such as, for example, non-compliance with the Protocol, etc.).

The withdrawal or discontinuation of the patient from the Study must be documented in the CRF. If a patient fails to return to the centre for a scheduled visit, attempts should be made to contact the patient to ensure that the reason for not returning is not a SAE. Likewise, if a patient declares his/her wish to discontinue from the study e.g. for personal reasons, an attempt should be made to establish that the true reason is not a SAE (bearing in mind the patient is not obliged to state his/her reasons).

Safety laboratory tests should be performed whenever possible at patient withdrawal.

It is also possible for the study to be terminated prematurely by the Sponsor in case of:

- new information leading to an unfavorable risk-benefit assessment of the IMP, such as occurrence of previously unknown significant adverse reactions or unexpectedly high intensity or incidence of known adverse reactions, or other unfavorable safety findings;
- Trial no longer justified for clinical or ethical reasons;
- withdrawal of IMP from the market for safety reasons.
- Finally, the trial could be terminated or suspended at the request of health authorities.

If the study will suffer of more than the expected 15% of dropout, the endpoints will be calculated anyway, as a proof of principle. In case of missing data to achieve the aims, these will be imputed using multiple imputation and a sensitivity analysis will be performed on the imputed data.

8.2.4. Lost to Follow-Up

A participant will be considered lost to follow-up if he or she repeatedly fails to return for scheduled visits and is unable to be contacted by the study site.

The following actions must be taken if a participant fail to return to the clinic for a required study visit:

- The site must attempt to contact the participant and reschedule the missed visit as soon as possible and counsel the participant on the importance of maintaining the assigned visit schedule and ascertain whether or not the participant wishes to and/or should continue in the study.
- Before a participant is deemed lost to follow-up, the investigator or designee must make every effort to regain contact with the participant (where possible, 3 telephone calls and,

if necessary, a certified letter to the participant's last known mailing address or local equivalent methods). These contact attempts should be documented in the participant's medical record.

- Should the participant continue to be unreachable, he/she will be considered to have withdrawn from the study with a primary reason of lost to follow-up.

8.3. Adverse Events

Adverse event (AE) evaluations will be performed during the whole duration of the study, starting from the time of signing of the Informed Consent and up to the end of the study.

8.3.1. Definitions

8.3.1.1. Adverse Event

An Adverse Event (AE) is defined as any untoward medical occurrence in a subject to whom a medicinal product is administered and which does not necessarily have a causal relationship with this treatment.
An AE can therefore be any unfavourable and unintended sign (including an abnormal laboratory finding), symptom, or disease temporally associated with the use of a medicinal product, whether or not related to the medicinal product.

8.3.1.2. Adverse Reaction

An Adverse Reaction (AR) is defined as a response to a medicinal product which is noxious and unintended and which occurs at doses normally used in man for the prophylaxis, diagnosis or therapy of disease or for the restoration, correction or modification of physiological function.
An AR can therefore be any unfavourable or unintended change in the structure (signs), function (symptoms), or chemistry (lab data) in a participant to whom an IMP has been administered and related to any dose administered. Medication errors, pregnancies and uses outside what is foreseen in the protocol, including misuse and abuse of the product, shall be subject to the same obligation to report as adverse reactions.

8.3.1.3. Serious Adverse Event

A serious adverse event (SAE) is defined as any untoward medical occurrence that at any dose requires inpatient hospitalisation or prolongation of existing hospitalisation, results in persistent or significant disability or incapacity, results in a congenital anomaly or birth defect, is life-threatening, or results in death.
An important medical event that based upon appropriate medical judgment, may jeopardize the patient and may require medical or surgical intervention to prevent one of the outcomes listed above is also an SAE.
Symptoms or medically significant laboratory or instrumental (e.g., electrocardiographic) abnormalities of a pre-existing disease, such as cancer or other disease, should not be considered an adverse event. However, occurrence of new symptoms, or laboratory or instrumental abnormalities, as well as worsening of pre-existing ones, are considered adverse events. Planned procedures that require hospitalization will not be considered SAEs.

8.3.1.4. Serious Adverse Reaction

A serious adverse reaction (SAR) is defined as an adverse reaction which results in death, is life-threatening, requires inpatient hospitalization or prolongation of existing hospitalization, results in persistent or significant disability or incapacity, or is a congenital anomaly/birth defect, or a medically important/clinically significant event.

8.3.1.5. Unexpected Adverse Drug Reaction
An unexpected adverse drug reaction is adverse event, the nature or severity of which is not consistent with the applicable product information (e.g., Investigator's Brochure for an unapproved investigational product or summary of product characteristics for an approved product).

8.3.2. Recording of adverse events
The Investigator is responsible for detecting, documenting, and reporting events that meet the definition of an AE or SAE.
Information on AEs should be obtained through observation of the patient, from any information volunteered by the patient, or through patient questioning.
All AEs (serious and non-serious) which occur from the signature of the informed consent until the final follow up visit will be recorded in the eCRF. The Investigator must provide information on the adverse event, preferably with a diagnosis, or at list with sign and symptoms; start and stop dates (and start and stop time if the adverse event lasts less than 24 hours); intensity; causal relationship to IMP; action taken and outcome.
Adverse events must be recorded individually, except when considered manifestations of the same medical condition or disease state; in such cases they must be recorded under single diagnosis. The Investigator must also provide start and stop dates (and start and stop time if the adverse event lasts less than 24 hours); intensity; causal relationship to IMP; action taken and outcome.
All AEs and SAEs must be followed until resolution, until the condition stabilizes, until the event is otherwise explained or until the patient completes the study or is lost to follow-up. The Investigator is responsible to ensure that follow-up includes any supplemental investigations as may be indicated to elucidate as completely as practical the nature and/or the causality of AE/SAE. This may include additional laboratory tests or investigations, or consultation with other health care professionals.

8.3.2.1. Seriousness of AE
When an AE occurs in a patient who has been administered at least one dose of the IMP, the Investigator responsible for the care of the subject must first assess whether the event is serious using the definition given in paragraph 8.3.1.3.
If the event is serious, then a Safety Report form must be completed and the CVBF Safety Management Team notified according to the procedure described in section 8.3.3.3.

8.3.2.2. Relationship of AE to the IMP
The Investigator will assess the possible relationship between the AE and the IMP, according to the following criteria:

- Reasonable possibility of a relatedness with study medication
- No reasonable possibility of relatedness with study medication

The expression "reasonable possibility of relatedness" is meant to convey in general that there are facts (evidence) or arguments that suggest a causal relationship. The Investigator will be also asked to consider the following before reaching a decision on causality assessment:

- time relationship between study drug intake and event's onset;
- medical history;
- lack of efficacy/worsening of existing condition;
- study treatment(s);
- mechanism of action of the investigational product;
- class effects;
- other treatments-concomitant or previous;
- withdrawal of study treatment(s);
- de-challenge (did the event abate after stopping drug?);

- re-challenge (did the event reappear after reintroduction?);
- erroneous treatment with study (or concomitant) medication;
- protocol related process.

8.3.2.3. Severity of AEs

Grading the intensity (severity) of a specific event is not the same as declaring it as "serious", which is based on patient/event outcome or action criteria usually associated with events that pose a threat to a patient's life or functioning. Seriousness (not severity) serves as a guide for defining regulatory reporting obligations.
The Investigator will grade the severity of all AEs using the definitions in the **Table below**. For each episode, the highest severity grade attained should be reported.

Mild	Grade 1 - Does not interfere with patient's usual function (awareness of symptoms or signs, but easily tolerated [acceptable]).
Moderate	Grade 2 - Interferes to some extent with patient's usual function (enough discomfort to interfere with usual activity [disturbing]).
Severe	Grade 3 - Interferes significantly with patient's usual function (incapacity to work or to do usual activities [unacceptable])

8.3.3. Serious Adverse Events Reporting

If an AE meets at least one seriousness criterion, this becomes an SAE and must be reported to the CVBF Safety Management Team as follows:

- The Investigator records the event in the patient's medical records and in the Adverse Event Form of the eCRF, indicating the seriousness of the event and the result of the causal relationship with the IMP assessment.
- The Investigator reports the SAE, regardless of presumed causal relationship, to CVBF Safety Management Team (delegated by the Sponsor) by e-mail (preferred) or fax within 24 hours of learning of the event. Contact details for SAE reporting are provided in the section "Study Personnel" of the protocol under Pharmacovigilance.

Expedited reporting (i.e., the notification within 24 hours of awareness of the event) must be carried out:

- from signature of informed consent up to the final follow-up visit (serious adverse event).
- with no time limitation if the event is likely to be related to the IMP (serious adverse reaction).

8.3.3.1. Notification process

Information on SAEs will be recorded in the Safety Report Form (SRF). Copy of the SRF will be archived in the Investigator's Site File. Follow-up reports (as many as required) should be completed and faxed/e-mailed following the notification procedure described below.
Whenever more than one SAE is observed, the Investigator should identify which is the primary adverse event, i.e. the most relevant one. If other events are listed in the same report, the Investigator, along with their relatedness to the IMP, should identify which adverse events are serious and which are non-serious. In any case, the Investigator is requested to record his/her opinion about the relatedness of the observed event(s) with the investigational medication.

Initial

The SRF must be completed by the Investigator or by his/her authorised delegate with all available information at the time of notification and then emailed or faxed to the CVBF Safety Management

Team within 24 hours of awareness of the event together with any extra annotated information/copies of test results.

Even if the Investigator has minimal information to include in the initial report (i.e., identifiable reporter, identifiable patient, a suspected investigational product, an adverse event), it is important that he/she always makes an assessment of causality to the IMP and/or concomitant medication and/or research event for every event prior to transmission of the SRF. The Investigator may change his/her opinion on causality in light of follow-up information and notify this change by sending a follow up SRF.

Follow-up

Patients must be followed-up until resolution of all queries, clinical recovery is complete and laboratory results have returned to normal baseline, or until progression has been stabilised, or until the patient is lost to follow-up. Follow-up may therefore continue until after the subject has left the study, if necessary.

Follow-up information should be recorded in a new SRF and sent by email/fax to the CVBF Safety Management Team as information becomes available. All relevant additional information (hospital discharge letter, blood analyses, etc.) should be attached to the SRF.

The subject must be identified by patient's ID and date of birth only. The patient's name should not be used on any correspondence and should be erased from any additional information provided.

8.3.3.2 Regulatory Reporting Requirements for SAEs

The CVBF Safety Management Team will review all SRFs received.

The causality assessment given by the Investigator will not be overruled and in case of disagreement, both opinions will be provided in any reports.

The CVBF Safety Management Team will comply with regulatory requirements relating to safety reporting to regulatory authorities and will also assure that all Investigators are informed of any safety issues that arise during the course of the trial.

All suspected unexpected serious adverse reactions (SUSARs), which occur with the investigational products within this clinical trial, will be submitted in compliance with the timelines and standards for reporting SUSARs set out in the Regulation (EU) no 536/2014 and linked guidance.

The concerned Regulatory Authorities will be informed via EudraVigilance and the Investigators by periodic line-listings.

8.3.4. Cardiovascular and Death Events

Not applicable.

8.3.5. Disease-Related Events and/or Disease-Related Outcomes Not Qualifying as AEs or SAEs

The following disease related events (DREs) are described in patients with Arrhythmogenic cardiomyopathy and can be serious/life threatening:

- Death for cardiac arrest.
- ICD intervention for VT.
- Cardiac-related syncope.
- Hospitalization for heart failure.

These events are rare, but typically associated with the disease under study. They will not be reported according to the standard process for expedited reporting of SAEs even though the event

may meet the definition of a SAE. These events will be recorded on the patient's eCRF within 3 days.

However, if either of the following conditions applies, then the event must be recorded and reported as an SAE (instead of a DRE):

- The event is, in the investigator's opinion, of greater intensity, frequency, or duration than expected for the individual patient.

OR

- The investigator considers that there is a reasonable possibility that the event was related to treatment with the investigational product.

8.3.6. Pregnancy

For female patients, information on pregnancies will be collected after the start of study treatment and up to 7 days after last dose or longer if requested by the DSMB.
If a pregnancy is reported, the Investigator should inform the CVBF Safety Management Team within 24 hours of learning of the pregnancy.
Abnormal pregnancy outcomes (e.g., spontaneous abortion, foetal death, stillbirth, congenital anomalies, ectopic pregnancy) are considered SAEs.

8.3.7. Code breaking

The randomization code can be unblinded upon written request to CCM, which retain the assignment correspondence. The date and reason that the blind was broken must be recorded in the source documentation and CRF, as applicable and referred to:

- the Investigators for emergency procedures.
- the CVBF Safety Management Team for pharmacovigilance purpose.

Individual treatment codes are kept in a secure location accessible only to designated staff in order to prevent dissemination of the treatment to personnel involved in study conduct who must remain blind.

8.3.7.1. Emergency procedures

The blinding should only be broken for valid medical or safety reasons, e.g., in case of a serious adverse event for which there is a reasonable possibility of a relatedness with the study medication (i.e., serious adverse reaction) and it is necessary for the PI or another delegated site staff member to know which treatment the patient is receiving before he/she can be properly treated for the event.
If the code is broken for an urgent safety situation, the patient will be withdrawn from the study.

8.3.7.2. Pharmacovigilance purposes

Treatment codes can only be broken by the CVBF Safety Management Team for the purpose of reporting a SUSAR to the regulatory authorities.

In the event of an SAE, the case is assessed by the Investigator for seriousness and causal relationship and by the CVBF Safety Management Team for expectedness.
If the case appears to be a SUSAR, then the blinding should be broken. It is important that the blind is being broken only for that specific patient.
Following unblinding,

a) if the product administered to the subject is atorvastatin, the serious adverse reaction will be reported as SUSAR to the regulatory authorities;

b) if the product administered to the subject is the placebo, the serious adverse reaction will be reassessed and if confirmed to be SUSAR (e.g., reaction due to an excipient or impurity), it will be reported to the regulatory authorities.

Suspected unexpected serious adverse reactions will be reported on expedite basis.

8.4. Treatment of Overdose

For this study, any dose of Atorvastatin greater than 240 mg within a 24-hour time period will be considered an overdose.

Decisions regarding dose interruptions or modifications will be made by the investigator in consultation with the Medical Monitor based on the clinical evaluation of the patient.

8.5. Safety Assessments

Planned time points for all safety assessments are provided in the SoA.

The most common (about 1:100) atorvastatin-related adverse reactions, based on data from clinical trials and extensive post-marketing experience are: allergic reactions, hyperglycemia, headache, pharyngolaryngeal pain, epistaxis, constipation, flatulence, dyspepsia, nausea, diarrhea, myalgia, arthralgia, pain in extremities, muscle spasms, joint swelling, back pain, abnormalities of liver function tests, increased creatine kinase, increased serum transaminases.

Uncommon (less than 1:100) adverse reactions include: loss of appetite, male gain, decreases in blood sugar levels, insomnia, dizziness, numbness or tingling in the fingers and toes, reductions of sensation to pain or touch, change in sense of taste, loss of memory, blurred vision, ringing in the ears and/or head, vomiting, belching, abdominal pain upper and lower, inflammations in pancreas or liver, skin rash and itching, hair loss, neck pain, muscle fatigue, chest pain, swelling especially in the ankles, raised temperature, urine tests that are positive for white blood cells.

Rare (1:1,000) adverse reactions include: serious allergic reaction, severe skin peeling, swelling or rash, Muscle weakness, pain, associated to red-brown discoloration of urine and high temperature (suggestive of rhabdomyolysis), visual disturbance, unexpected bleeding or bruising cholestasis (yellowing of the skin and whites of the eyes), tendon injury.

Very rare (1:10,000) adverse reactions include: Lupus-like disease syndrome (including rash, joint disorders and effects on blood cells); unexpected or unusual bleeding or bruising, suggestive of a liver complaint, hearing loss, gynecomastia.

The parameters used to ensure safety are mainly constituted by blood tests and patient reported adverse events.

Enrolled patients will be encouraged to report adverse events spontaneously even without direct questioning by the investigator. For each adverse event reported by the patient, the investigator will obtain all information and documentation necessary for an accurate description of the adverse event.

The adverse events can be reported by the patient at any time, as soon as possible during-after occurrence. Blood tests at the visits T2, T4,T9 and T18 might uncover some hidden adverse events.

All adverse events, regardless of severity, or presumed relationship to the experimental intervention or control intervention planned for the study will be recorded in the clinical record.

The record will include: level of severity, start and end date, drug correlation assessment and measures taken to address the adverse event, final outcome.

8.5.1. Physical Examinations

A brief physical examination will include assessments of the cardiovascular system and abdomen (liver and spleen). Height and weight will also be measured and recorded.

8.5.2. Vital Signs

Pulse rate and blood pressure will be assessed. Blood pressure and pulse measurements should be preceded by at least 5 minutes of rest for the participant in a quiet setting without distractions (eg, television, cell phones). In addition, physical exercise habits will be registered.

8.5.3. Electrocardiograms

12-lead ECG will be obtained as outlined in the SoA.

8.5.4. Clinical Safety Laboratory Assessments

The list of clinical laboratory tests to be performed are provided in the SoA together with the timing and frequency.

The investigator must review the laboratory report, document this review, and record any clinically relevant changes occurring during the study in the AE section of the CRF. The laboratory reports must be filed with the source documents. Clinically significant abnormal laboratory findings are those which are not associated with the underlying disease, unless judged by the investigator to be more severe than expected for the participant's condition.

8.5.5. Genetics

ACM patients who have not yet undergone genetic testing will be analyzed by University of Naples Federico II, by whole-exome sequencing. The whole exome will be captured with Agilent SureSelect Human All Exome V6 and each genomic DNA will be univocally tagged with a barcode sequence to allow samples multiplexing during the subsequent sequencing step. The obtained enriched and indexed libraries will be sequenced on an Illumina NovaSeq 6000 System. The results of the analysis of the genes associated to ACM, as for Expert Consensus Statement on the state of genetic testing for cardiac diseases [29], will be recorded in the CRF and used to stratify the patients.

8.5.6. Biomarkers

Biomarkers will comprise those to determine the safety of the IMP and those which could determine or represent ACM progression, and could eventually influence Atorvastatin effect. They will be searched in plasma or serum.

Beta-HCG, haemochrome, LDL, NTpro BNP, Troponin, CRP, Interleukin6, ST2, CK, AST/ALT, ALP, Bilirubin, LDH, gammaGT, Creatinine, Timp1, PIIINP, will be measured by the diagnostic laboratories of the different centers, using clinical-grade kits. Biomarkers for which standard diagnostic procedures are not on the market or are not usually available in standard clinical labs, will be centrally measured by the research laboratory of Centro Cardiologico Monzino IRCCS, in order to standardize the protocol, with available research-grade Elisa kits. Those includes oxLDL, MDA, 4HNE, Interleukin-6, TNF alfa, TGFβ, BIN1, GAL3, HSP70, estradiol, testosterone. See Annex V for a complete list.

8.5.7. Metabolomic Research

To further dissect the specificity of Atorvastatin on particular lipid pathways, a specific lipidomic signature will be defined in patients at the baseline and in response to the treatment. This will enclose the characterization of several lipid classes, including di- and triglycerides, acylcarnitines, cholesteryl esters ceramides, etc [30]. The definition of a set of dysregulated lipids whose abundance may be rescued after drug administration will provide a collection of analytes with predictive value, providing a description of the metabolic disruptions/restoration. The lipidomic characterization of samples will be performed by University of Naples Federico II, employing the standardized protocols of the MxP® Quant 500 XL kit (Biocrates Life Sciences Innsbruck, Austria), in combination with MS analysis. The kit will allow the analysis of roughly 80 real samples per plate, providing the quantification of more than 1,000 metabolites from various biochemical

classes, with more than 400 quantifiable metabolism indicators covering hundreds of metabolic pathways.

9. Statistical Considerations

9.1. Sample Size Determination

A power analysis estimated that 88 patients (44 per group) will be sufficient to observe at T18 a significant (alpha=0.05) mean absolute difference between the two groups of 2.4 points of RV free wall longitudinal strain (%), hypothesizing a standard deviation of 4.5% and with a power of 80%. The statistical test used to calculate the sample size was an independent one-sided T test. Assuming a drop-out rate of 15% during the study, the total sample size is 102 patients (51 per group). Assuming a screening failure of about 5%, 110 patients will be screened.

9.2. Populations for Analyses

The Safety population will consist of all patients who received any study medication and will be based on the treatment actually received. The Safety population will be used to present the demographic and baseline data, and all safety data.

The Intent to Treat (ITT) population will consist of all patients who are randomized and receive the IMP (either Atorvastatin or placebo); it will be based on the treatment randomized, regardless of the treatment actually received. The ITT population will be used to present efficacy data.

9.3. Statistical Analyses

All patient data collected on the CRF will be listed by patient and centre.

The Statistical Analysis Plan, describing details of all the statistical methods and analysis to be applied to trial results is described below Any change in the planed analysis will be documented. The data will be presented in the clinical study report.

Continuous variables will be reported as mean ± standard deviation (SD) if normally distributed, otherwise as median and interquartile range (IQR). For variables with right-skewed distribution, a log-transformation will be considered before analysis, otherwise nonparametric methods will be considered. Categorical variables will be presented as frequencies and percentages. All analyses will be performed using SAS version 9.4 (SAS Institute, Cary, NC, USA).

Possible differences in baseline characteristics of ACM patients belonging to the “Atorvastatin group” and the “placebo group” will be reported in a descriptive manner.

Changes in the variables of interest, measured at different timepoints, will be assessed by paired samples Student's T test or Wikcoxon signed-rank test, and will be compared between groups by Student's T test for independent samples or Mann Whitney's U test.

Differences of treatment effects on the primary and secondary endpoints in the groups will be analysed by using univariate and multivariate regression models, also taking into account possible confounding variables.

Data on the primary endpoint measures will also be available for most subjects before baseline (extended baseline). The analyses will apply time series models to compare the two groups (focusing on rate of change).

Subgroup analyses will account for other medications, underlying genetic mutations or disease stage.

As proof of mechanism, Person's or Spearman's correlation coefficients will be performed to assess possible relationships between endpoints and plasma oxLDL reduction.

Data from the psychological questionnaires will be analyzed by repeated-measures ANOVA models, in order to analyses the variables at different timepoints. For within-patient comparisons, paired sample t-tests will be implemented. Adjustments for possible confounding variables will be considered.

For the third objective, different AI models will be created to exploratively assess the association between a large number of potential predictors (below referred as variables) and the response to treatment, in terms of primary and secondary study endpoints. In this case, the test cohort will be

the “Atorvastatin Group”. Standard clinical variables and symptoms, together with biomarkers (as plasma lipids, inflammatory, cardiac damage and fibrosis markers), genotype and image-derived features (extracted from ECHO and CMR), collected at T0, will be taken into account as possible predictive variables to define the models. In particular, image-derived features will be obtained by radiomics analysis, which extracts a large number of quantitative characteristics that fail to be detected by the naked eye. Different models will be developed to describe the association of the previously described variables, either as single domains or combined, with the endpoint outcomes at T18 of the patients receiving Atorvastatin. Several feature selections (e.g. filter and wrapper methods) and AI algorithms (e.g. random forest, support vector classifier, k-nearest neighbors) will be used to develop multi-modal models for the prediction of response to Atorvastatin. In view of the relatively small sample size involved, a nested cross-validation approach will be used to overcome test set selection bias. The final model will be obtained by re-training the best performing pipeline in nested cross-validation on the entire dataset, with hyperparameter values tuned with a regular k-fold cross-validation on the whole dataset. Performance metrics will include balanced accuracy, sensitivity, specificity and f1 score.

9.1.1 Demographic and baseline characteristics

Demographic and baseline characteristics will be summarized for all patients in the Safety population, by treatment group.

9.1.2 Analysis of efficacy variables

SEARCH TRIAL first aim is to provide evidence of Atorvastatin clinical efficacy in preventing ACM deterioration. The variable used to determine efficacy in the primary endpoint is the progressive decline of RV free wall longitudinal strain measured by ECHO at T18.

The variables used to determine efficacy in the secondary endpoint at T18 are i) arrhythmia burden (PVC, non-sustained and sustained VA, VF, appropriate ICD shocks); ii) other morphological parameters (ventricular volumes, function, both at ECHO and CMR); iii) ECG parameters; iv) blood parameters.

9.1.3 Analysis of safety variables

AEs will be presented in terms of the number of AE, the incidence, severity and relationship to the study drug, overall and by body system and preferred term. SAEs will be presented in the same way.

Results for routine laboratory tests will be assessed as being below the lower limit of the normal range, within the normal range or above the upper limit of the normal range. The frequency of patients reporting an abnormal or abnormal clinically significant laboratory value at screening and follow-up will be presented for each laboratory variable. Vital signs will be presented using descriptive statistics.

9.1.4 Missing data

All reasonable efforts will be made to reduce the rate of missing data, since any method used for imputation for missing observations would be based on untestable assumptions.

In case of impossibility of performing CMR in some patients, the patients will be excluded from the analysis of the specific elements, based on CMR, of the secondary endpoint. The same parameters acquired by ECHO will be used.

Investigators will be trained about the importance of patient retention and full data capture. Also, any reasonable attempts should be made by the Investigators to emphasize continued patient’s participation for the full duration of the trial. However, in order to minimize missing data, if a patient cannot refer to the site for a planned follow-up visit, the Investigator will try to obtain any relevant

information from the patients, including documents/laboratory results available from local medical care.
However, if a patient is lost at follow-up after the T9 data collection visit, imputation of missing data may be implemented, when possible, followed by sensitivity analyses.
If the patient drops-out before T9 the patient will be simply excluded from the analysis.
We expect that a larger number of patients will abandon the study if they are in the Atorvastatin group, respect to the Placebo one, since they have probability to experience atorvastatin-related adverse effects. The 15% drop-out rate described in the sample size calculations, takes into account this possibility.
An effort will be made to understand the reasons of drop-outs, mainly to assess whether they are related to the outcomes (ACM-linked events). In this case, to avoid a possible bias (the most serious patients abandon the study) a post-hoc statistical correction may be implemented.
We will try to reduce unused data (collected data items that will not directly link to a result and will therefore not be counted in the final manuscript), and spurious data or spurious inferences, to improve study performance, lower time and costs of collection and storage, and address ethical concerns.

10. Ethical Considerations

The study is conducted in accordance with the ethical principles expressed by the Helsinki Declaration (1964), Summerset West, South Africa (1996) amendment and with the international standards of Good Clinical Practice, ICH (International Conference on Harmonization) and in accordance with all local laws and regulations concerning clinical studies.

10.1. Independent Ethics Committee (IEC)

It is the responsibility of CCM to obtain approval of the trial protocol/amendments from the appropriate IEC.

Prior to the initiation of the study, the followings will be submitted to the IEC for approval:

- the study protocol,
- the ICF,
- Investigators' current curriculum vitae,
- Insurance certificate
- any other requested document(s).

A copy of the IEC approval will be sent to CCM, along with all other correspondence with the IEC, including the submission documents. The Investigator should file copies of relevant IEC documents in the Investigator Site File.

The study will not be started until full written approval has been obtained from the appropriate IEC. The letter of approval should be dated, and should specify the type (e.g. protocol number) and the date of the documents which were reviewed and approved.

CCM will submit any future amendment to the protocol to the IEC which granted the original approval. Any amendment will be implemented only when full approval has been obtained from the appropriate IEC, except for those amendments which involve only logistical or administrative aspects of the study.

CCM will submit required progress reports to the IEC which approved the protocol at least annually, as well as report any serious ADRs, life-threatening problems or deaths.

CCM will also inform the IEC of reports of serious ADRs occurred at other sites participating to this clinical trial.

CCM must inform the IEC of the termination of the study.

10.2. Informed consent

The Investigator, or a person designated by the Investigator, will provide the patients with the written Informed Consent Form (ICF). It is the responsibility of the Investigators to obtain written informed consent from the participants before inclusion in the trial, after an exhaustive explanation of the nature, objectives and risks inherent in participating in it. Participants must be informed that participation is voluntary. The patient will be required to sign written consent that meets the requirements of 21 CFR 50, local regulations, International Conference on Harmonisation (ICH) guidelines, Health Insurance Portability and Accountability Act (HIPAA) requirements, where applicable, and the IRB/IEC or study center after the nature of the study has been fully explained and before performance of any study-related activity.

- The medical record must include a statement that written informed consent.
- Participants must be re consented and re-assented to the most current version of the ICF(s) during their participation in the study.
- A copy of the informed consent and assent forms must be provided to the participant.
- It will be possible to withdraw the consent at any time and/or request the elimination of biological material obtained for research purposes at any time.

- Stored samples will be coded throughout the sample storage and analysis process and will not be labelled with personal identifiers. Participants may withdraw their consent for their samples to be stored for research.

10.3. Insurance

Before the trial formally starts, CCM will take out a study-specific insurance covering the amount requested by the respective national laws for patients/Investigators/Institutions participating in the clinical trial.
Insurance and any updates will be provided to the Investigator before trial commencement for filing into the Investigator Site File.
For risks arising from participation in the study, CCM has adequate insurance coverage stipulated following the relevant laws in force. By contract, the insurance will only respond in cases where the damage suffered by the subject is related to the treatment or any procedure associated with the participation in the study. The insurance will not reimburse damages resulting from procedures conducted by the subject not covered by this protocol.
In case of questions about medical care, cost for medical care or insurance, patients can talk to their Investigator. Contact details will be given in the ICF.

10.4. Confidentiality and protection of Personal Data

10.4.1 Confidentiality

Confidentiality will be assured for the whole duration of the study. By signing the protocol, the PI agrees to keep all information related to the study in strict confidence and to request similar confidentiality from his/her staff.. All records and study documents (Protocol, Investigator's Brochures and other material) will be kept in a locked file cabinet. All computer entries will be done using the subject ID only.
The information provided by the Sponsor to the PI may not be disclosed to others without direct written authorisation from the Sponsor, except to the extent necessary to obtain informed consent from patients.
An agreement for disclosure will be obtained in writing by the patient and will be included in the ICF. All information obtained during the conduct of the study will be regarded as confidential.

10.4.2 Protection of Personal Data

All measures will be taken to ensure the data protection of patients participating in this study. Patient's data collected during the study and, generally, the study procedures are compliant with the General Data Protection Regulation (EU) 2016/679.

Patients will be informed of their rights under Art. 15 et seq. of the GDPR (right of access, correction, blocking, deletion or cancellation), including the withdrawal of their consent.
On the CRFs patients will be identified ONLY by the assigned patient number. If patient names are included on copies of documents required for the study the names will be obliterated or masked and the assigned patient number added to the document.
The Investigator should keep a separate log (Patient Master List) of patient's codes, names and addresses.Will be adopted appropriate and preventive security measures to minimize the risks of destruction or loss, even accidental, of personal data, unauthorized access or treatment that is not permitted or does not comply with the purposes of the collection
Clinical information will not be released without written permission of the patients, except as necessary for monitoring by IEC, the sponsor or the sponsor's designee.

For the entire duration of the study, all Personal Data made available and/or developed in the course of the study and in pursuit of its objectives will be kept confidential adopting any measure

(of contractual, technological or physical nature) suitable for their protection, including towards its own employees, collaborators, sub-contractors, assigns or successors in title.

10.4.2.1 Responsibility regarding the processing of personal data

The data holders are:

- Centro Cardiologico Monzino, IRCCS which has its business office in Via C. Parea, 4 - 20138 Milan, for the processing of the personal data of all patients participating in the project, in particular for the storage of data in special databases and for the elaboration and analysis and production of reports.
- Each center for the processing of personal data of its patients participating in the project.

10.4.3 Dissemination of clinical study data

Once the study has been completed and all the resulting information collected, data will be analysed to draw conclusions. The Sponsor is responsible to make them available to the scientific community (e.g. journal publications, trial registry, trial website).
Under the terms of the Declaration of Helsinki, the International Council for Harmonisation E6 Good Clinical Practice Consolidated Guideline, and all applicable laws and regulations, including, without limitation, data privacy laws, clinical trial disclosure laws, and Regulation (EU) No 536/2014, the clinical trial results will be made public through the European portal for the management and conduct of clinical trial (https://euclinicaltrials.eu) and/or any of the WHO Primary Registries. Before the start of any clinical trial its details must be entered into a publicly accessible trial register in accordance with international standards defined by WHO. Registration of the trial should occur before the first intervention is administered to the first participant.

10.5. Publication Policy

Results of the study will be published on internationally recognized and impacted scientific journals characterized by an open-access policy. Authorships in all publications will be consistent with academic standards. Raw data underlying the publications will be deposited in open-access public repositories. In each publication, express mention will be made of the funding provided (PNRR-MCNT2-2023-1237697812376978), the CUP code identifying the project and the EU emblem. In addition, a communication to the founding agency will be made.

11. Data Handling and Record Keeping

11.1. Case Report Form

CRFs are property of CCM and should not be made available in any form to third parties (except for authorized CCM designee or representatives of appropriate Health/Regulatory Authorities) without written permission from CCM.

The Investigator will be responsible for the accuracy of the data entered in the CRFs. All entries must be written in *ENGLISH*. Source documents should be available to support all the data recorded in the CRF; location of source documents, including those for which the CRF might be accepted as being the sole source document, will be specified and listed in an appropriate document.

The CRF must be available for review/collection to designated CCM representatives at each scheduled monitoring/audit visit.

11.2. Data management

Data management of the CRFs will be performed by CCM.

Once all data queries have been resolved, the study will be declared to be “clean”, and the study data will be locked ready for analysis.

12. Study Management

The study will be performed in accordance with the protocol, the Declaration of Helsinki (64th WMA General Assembly, Fortaleza, Brazil, October 2013) and ICH Harmonised Tripartite Guideline for Good Clinical Practice (ICH-GCP) and any local regulations.

12.1. Monitoring

Monitoring will be carried out by CRAs appointed by CVBF.
The purpose of the monitoring visit is to verify that the rights and the wellbeing of the patient are protected, that the reported data are accurate, complete and verifiable from source documents and that the conduct of the trial complies with the currently approved protocol and any amendments, with ICH GCP, and with regulatory requirements.
Prior to study start, the Investigator will be informed of the anticipated frequency of the monitoring visits. (S)He will also receive a notification prior to each monitoring visit during the course of the study. It is expected that the Investigator and/or his/her sub-Investigator(s) and other appropriate staff will be available on the day of the visit to discuss study conduct and to cooperate with the monitor to ensure that any problems detected during the course of these monitoring visits are resolved.

12.2. Audit and Inspection

Audit of appropriate documents will be performed by the Sponsor Quality Assurance (or designee).
The study site may be audited by a regulatory agency on one or more occasions. The Investigators will be informed in advance of such a visit.

12.3. Protocol Deviations/amendments

Changes to the Protocol will be implemented only when written amendments have been signed by all individuals who signed the protocol.
Any amendment will be sent to the IEC and Competent Authority as appropriate. No deviations from or changes to the protocol will be implemented without documented approval of an amendment from the IEC which granted the original approval, except where necessary to eliminate an immediate hazard(s) to trial patient, or when the change(s) involves only logistical or administrative aspects of the trial. The deviations from or changes to the protocol implemented to eliminate an immediate hazard to the trial patient and the proposed amendment, if appropriate, should be submitted to the IEC for review and approval as soon as possible.
Any other deviation from the protocol that has not been approved by CCM and the IEC could result in a discontinuation from the study at the centre involved.
Any written amendment will be sent to all recipients of the protocol.

13. References

1. Sommariva, E.; Stadiotti, I.; Casella, M.; Catto, V.; Dello Russo, A.; Carbucicchio, C.; Arnaboldi, L.; De Metrio, S.; Milano, G.; Scopece, A., et al. Oxidized LDL-dependent pathway as new pathogenic trigger in arrhythmogenic cardiomyopathy. *EMBO Mol Med* **2021**, *13*, e14365, doi:10.15252/emmm.202114365.
2. Marcus, F.I.; McKenna, W.J.; Sherrill, D.; Basso, C.; Bauce, B.; Bluemke, D.A.; Calkins, H.; Corrado, D.; Cox, M.G.; Daubert, J.P., et al. Diagnosis of arrhythmogenic right ventricular cardiomyopathy/dysplasia: proposed modification of the Task Force Criteria. *Eur Heart J* **2010**, *31*, 806-814, doi:10.1093/eurheartj/ehq025.
3. Corrado, D.; Perazzolo Marra, M.; Zorzi, A.; Beffagna, G.; Cipriani, A.; Lazzari, M.; Migliore, F.; Pilichou, K.; Rampazzo, A.; Rigato, I., et al. Diagnosis of arrhythmogenic cardiomyopathy: The Padua criteria. *Int J Cardiol* **2020**, *319*, 106-114, doi:10.1016/j.ijcard.2020.06.005.
4. Mach, F.; Baigent, C.; Catapano, A.L.; Koskinas, K.C.; Casula, M.; Badimon, L.; Chapman, M.J.; De Backer, G.G.; Delgado, V.; Ference, B.A., et al. 2019 ESC/EAS Guidelines for the management of dyslipidaemias: lipid modification to reduce cardiovascular risk. *Eur Heart J* **2020**, *41*, 111-188, doi:10.1093/eurheartj/ehz455.
5. Muthappan, P.; Calkins, H. Arrhythmogenic right ventricular dysplasia. *Prog Cardiovasc Dis* **2008**, *51*, 31-43, doi:10.1016/j.pcad.2008.01.002.
6. Hulot, J.S.; Jouven, X.; Empana, J.P.; Frank, R.; Fontaine, G. Natural history and risk stratification of arrhythmogenic right ventricular dysplasia/cardiomyopathy. *Circulation* **2004**, *110*, 1879-1884, doi:10.1161/01.CIR.0000143375.93288.82.
7. Basso, C.; Corrado, D.; Marcus, F.I.; Nava, A.; Thiene, G. Arrhythmogenic right ventricular cardiomyopathy. *Lancet* **2009**, *373*, 1289-1300, doi:10.1016/S0140-6736(09)60256-7.
8. Maron, B.J.; Thompson, P.D.; Ackerman, M.J.; Balady, G.; Berger, S.; Cohen, D.; Dimeff, R.; Douglas, P.S.; Glover, D.W.; Hutter, A.M., Jr., et al. Recommendations and considerations related to preparticipation screening for cardiovascular abnormalities in competitive athletes: 2007 update: a scientific statement from the American Heart Association Council on Nutrition, Physical Activity, and Metabolism: endorsed by the American College of Cardiology Foundation. *Circulation* **2007**, *115*, 1643-1455, doi:10.1161/CIRCULATIONAHA.107.181423.
9. Tabib, A.; Miras, A.; Taniere, P.; Loire, R. Undetected cardiac lesions cause unexpected sudden cardiac death during occasional sport activity. A report of 80 cases. *Eur Heart J* **1999**, *20*, 900-903, doi:10.1053/euhj.1998.1403.
10. Thiene, G.; Nava, A.; Corrado, D.; Rossi, L.; Pennelli, N. Right ventricular cardiomyopathy and sudden death in young people. *N Engl J Med* **1988**, *318*, 129-133, doi:10.1056/NEJM198801213180301.
11. Corrado, D.; Wichter, T.; Link, M.S.; Hauer, R.N.; Marchlinski, F.E.; Anastasakis, A.; Bauce, B.; Basso, C.; Brunckhorst, C.; Tsatsopoulou, A., et al. Treatment of Arrhythmogenic Right Ventricular Cardiomyopathy/Dysplasia: An International Task

Force Consensus Statement. *Circulation* **2015**, *132*, 441-453, doi:10.1161/CIRCULATIONAHA.115.017944.

12. Migliore, F.; Mattesi, G.; Zorzi, A.; Bauce, B.; Rigato, I.; Corrado, D.; Cipriani, A. Arrhythmogenic Cardiomyopathy-Current Treatment and Future Options. *J Clin Med* **2021**, *10*, doi:10.3390/jcm10132750.

13. Davignon, J.; Jacob, R.F.; Mason, R.P. The antioxidant effects of statins. *Coron Artery Dis* **2004**, *15*, 251-258, doi:10.1097/01.mca.0000131573.31966.34.

14. Malik, N.; Win, S.; James, C.A.; Kutty, S.; Mukherjee, M.; Gilotra, N.A.; Tichnell, C.; Murray, B.; Agafonova, J.; Tandri, H., et al. Right Ventricular Strain Predicts Structural Disease Progression in Patients With Arrhythmogenic Right Ventricular Cardiomyopathy. *J Am Heart Assoc* **2020**, *9*, e015016, doi:10.1161/JAHA.119.015016.

15. Anwer, S.; Stollenwerk, L.; Winkler, N.E.; Guastafierro, F.; Hebeisen, M.; Akdis, D.; Saguner, A.M.; Brunckhorst, C.; Duru, F.; Tanner, F.C. Right heart strain in arrhythmogenic right ventricular cardiomyopathy: Implications for cardiovascular outcome. *Eur Heart J Cardiovasc Imaging* **2024**, 10.1093/ehjci/jeae117, doi:10.1093/ehjci/jeae117.

16. Kirkels, F.P.; van Osta, N.; Rootwelt-Norberg, C.; Chivulescu, M.; van Loon, T.; Aabel, E.W.; Castrini, A.I.; Lie, O.H.; Asselbergs, F.W.; Delhaas, T., et al. Monitoring of Myocardial Involvement in Early Arrhythmogenic Right Ventricular Cardiomyopathy Across the Age Spectrum. *J Am Coll Cardiol* **2023**, *82*, 785-797, doi:10.1016/j.jacc.2023.05.065.

17. Goldberger, J.J.; Subacius, H.; Schaechter, A.; Howard, A.; Berger, R.; Shalaby, A.; Levine, J.; Kadish, A.H.; Investigators, D. Effects of statin therapy on arrhythmic events and survival in patients with nonischemic dilated cardiomyopathy. *J Am Coll Cardiol* **2006**, *48*, 1228-1233, doi:10.1016/j.jacc.2006.05.053.

18. Sinagra, G.; Cappelletto, C.; A, D.E.L.; Romani, S.; Paldino, A.; Korcova, R.; Ferro, M.D.; Vitrella, G.; Pagnan, L.; Pinamonti, B. Focus on arrhythmogenic right ventricular cardiomyopathy. *Eur Heart J Suppl* **2020**, *22*, L129-L135, doi:10.1093/eurheartj/suaa152.

19. Beffagna, G.; Sommariva, E.; Bellin, M. Mechanotransduction and Adrenergic Stimulation in Arrhythmogenic Cardiomyopathy: An Overview of in vitro and in vivo Models. *Front Physiol* **2020**, *11*, 568535, doi:10.3389/fphys.2020.568535.

20. Aydin, M.U.; Aygul, N.; Altunkeser, B.B.; Unlu, A.; Taner, A. Comparative effects of high-dose atorvastatin versus moderate-dose rosuvastatin on lipid parameters, oxidized-LDL and inflammatory markers in ST elevation myocardial infarction. *Atherosclerosis* **2015**, *239*, 439-443, doi:10.1016/j.atherosclerosis.2015.02.003.

21. Ky, B.; Burke, A.; Tsimikas, S.; Wolfe, M.L.; Tadesse, M.G.; Szapary, P.O.; Witztum, J.L.; FitzGerald, G.A.; Rader, D.J. The influence of pravastatin and atorvastatin on markers of oxidative stress in hypercholesterolemic humans. *J Am Coll Cardiol* **2008**, *51*, 1653-1662, doi:10.1016/j.jacc.2008.01.026.

22. Bybee, K.A.; Lee, J.H.; O'Keefe, J.H. Cumulative clinical trial data on atorvastatin for reducing cardiovascular events: the clinical impact of atorvastatin. *Curr Med Res Opin* **2008**, *24*, 1217-1229, doi:10.1185/030079908x292001.

23. Newman, C.; Tsai, J.; Szarek, M.; Luo, D.; Gibson, E. Comparative safety of atorvastatin 80 mg versus 10 mg derived from analysis of 49 completed trials in 14,236 patients. *Am J Cardiol* **2006**, *97*, 61-67, doi:10.1016/j.amjcard.2005.07.108.

24. Marcus, F.I.; McKenna, W.J.; Sherrill, D.; Basso, C.; Bauce, B.; Bluemke, D.A.; Calkins, H.; Corrado, D.; Cox, M.G.; Daubert, J.P., et al. Diagnosis of arrhythmogenic right ventricular cardiomyopathy/dysplasia: proposed modification of the task force criteria. *Circulation* **2010**, *121*, 1533-1541, doi:10.1161/CIRCULATIONAHA.108.840827.

25. Arbelo, E.; Protonotarios, A.; Gimeno, J.R.; Arbustini, E.; Barriales-Villa, R.; Basso, C.; Bezzina, C.R.; Biagini, E.; Blom, N.A.; de Boer, R.A., et al. 2023 ESC Guidelines for the management of cardiomyopathies. *Eur Heart J* **2023**, *44*, 3503-3626, doi:10.1093/eurheartj/ehad194.

26. Steinberg, J.S.; Joshi, S.; Schron, E.B.; Powell, J.; Hallstrom, A.; McBurnie, M.; Investigators, A. Psychosocial status predicts mortality in patients with life-threatening ventricular arrhythmias. *Heart Rhythm* **2008**, *5*, 361-365, doi:10.1016/j.hrthm.2007.11.010.

27. Zigmond, A.S.; Snaith, R.P. The hospital anxiety and depression scale. *Acta Psychiatr Scand* **1983**, *67*, 361-370, doi:10.1111/j.1600-0447.1983.tb09716.x.

28. Creamer, M.; Bell, R.; Failla, S. Psychometric properties of the Impact of Event Scale - Revised. *Behav Res Ther* **2003**, *41*, 1489-1496, doi:10.1016/j.brat.2003.07.010.

29. Wilde, A.A.M.; Semsarian, C.; Marquez, M.F.; Shamloo, A.S.; Ackerman, M.J.; Ashley, E.A.; Sternick, E.B.; Barajas-Martinez, H.; Behr, E.R.; Bezzina, C.R., et al. European Heart Rhythm Association (EHRA)/Heart Rhythm Society (HRS)/Asia Pacific Heart Rhythm Society (APHRS)/Latin American Heart Rhythm Society (LAHRS) Expert Consensus Statement on the state of genetic testing for cardiac diseases. *Europace* **2022**, *24*, 1307-1367, doi:10.1093/europace/euac030.

30. Costanzo, M.; Caterino, M. Targeted lipidomics data of COVID-19 patients. *Data Brief* **2023**, *48*, 109089, doi:10.1016/j.dib.2023.109089.

14. Annexes List

Annex I: CTFG - Recommendations related to contraception and pregnancy testing in clinical trials

Annex II: Packaging and labelling details

Annex III: Drug Accountability

Annex IV: Psicological questionnaries (Annex IVa: HADS; Annex IVb: IES-R; Annex IVc: SF36)

Annex V: Clinical Laboratory Tests
